# Exploring the potential biological significance of KDELR family genes in lung adenocarcinoma

**DOI:** 10.1038/s41598-024-65425-2

**Published:** 2024-06-27

**Authors:** Peitong Li, Pengfei Cui, Qing Yue, Zijun Xu, Ziling Liu

**Affiliations:** Cancer Center, The First Hospital of Jilin University, Changchun, China

**Keywords:** Cancer, Computational biology and bioinformatics

## Abstract

The Lys-Asp-Glu-Leu receptor (KDELR) family genes play critical roles in a variety of biological processes in different tumors. Our study aimed to provide a comprehensive analysis of the potential roles of KDELRs in lung adenocarcinoma (LUAD). Utilizing data from The Cancer Genome Atlas (TCGA) and Gene Expression Omnibus (GEO) database, as well as clinical samples, we conducted a series of analyses and validations using R software tools and various online resources. The results showed that KDELR family genes and proteins were highly expressed and associated with a poor prognosis of LUAD. Promoter hypomethylation and the competing endogenous RNA (ceRNA) network of PCAT6/hsa-miR-326/KDELR1 might be potential causes of aberrant KDELR1 overexpression in LUAD. Three key Transcription factors (TFs) (SPI1, EP300, and MAZ) and a TFs-miRNAs-KDELRs network (involving 11 TFs) might be involved in modulating KDELRs expression abnormalities. Gene Set Enrichment Analysis (GSEA) indicated enrichment of genes highly expressing KDELR1, KDELR2, and KDELR3 in MTORC1_SIGNALING, P53_PATHWAY, and ANGIOGENESIS. Negative correlations between KDELRs expression and CD8 + T cell infiltration, as well as CTLA-4 expression. Our multiple analyses suggested that the KDELRs are important signaling molecules in LUAD. These results provided novel insights for developing prognostic markers and novel therapies of LUAD.

## Introduction

Lung cancer accounts for the highest percentage of cancer deaths, with 1.8 million annual deaths recorded globally in recent years^1,2^. Non-small cell lung cancer (NSCLC), the predominant subtype, constitutes approximately 80% of all lung cancer cases, with lung adenocarcinoma (LUAD) being the prevailing histological subtype within NSCLC^1^. Despite notable advancements in the molecular pathology and targeted therapeutic strategies for LUAD, the 5-year overall survival (OS) rate for this malignancy remains dismally low at around 15%^3^. Given the low cure rate and high recurrence rate, a comprehensive understanding of the molecular underpinnings driving the initiation and progression of LUAD is essential for early detection, precision treatment, and prognosis monitoring.

The Lys-Asp-Glu-Leu receptor (KDELR), a member of the PQ cyclin family, recognizes and binds to the KDEL-like motif at the C-terminal of endoplasmic reticulum (ER) chaperonins (also known as ER proteins), and backtracks from Golgi to the ER via Coat Protein I (COPI) vesicles. This maintains the dynamic balance between the Golgi and the ER^4,5^. This was one of the first physiological roles of KDELRs to be discovered. Later, with further research, KDELRs were found to be pivotal regulators in cellular trafficking, immunity and autophagy^6–8^. Notably, recent findings suggest that KDEL receptors have a Golgi-retention function, acting as Golgi-gatekeepers^9^. In addition to traveling between the Golgi and ER, KDEL receptors also circulate between the Golgi and the plasma membrane, contributing to the cell surface binding of ER proteins, thereby fostering cell proliferation and migration^10,11^. Further exploration revealed that KDELR, as a receptor for ER proteins secreted at the cell membrane, has its own downstream signaling network, including trans-activation of the EGFR-STAT3 signaling pathway^12^.

Moreover, accumulating evidence have shown that KDELRs are associated with the development and progression of different kinds of tumors. For example, KDELR1 deficiency enhanced melanoma metastasis^13^, while activation of KDELR2 triggered HDAC3-mediated promotion of breast cancer proliferation^14^. KDELR2 also harnessed the mTOR signaling pathway to promote glioblastoma tumorigenesis^15^, and was implicated in unfavorable prognostic markers for gliomas^16^. It was demonstrated that KDELR2 acted as a driver of lung cancer invasion and metastasis^17^. KDELR3 was identified to be involved in promoting melanoma metastasis^13^. However, a systematic examination of the entire KDELR family in tumors, including LUAD, remains conspicuously absent in the extant literature.

**Figure 1:**
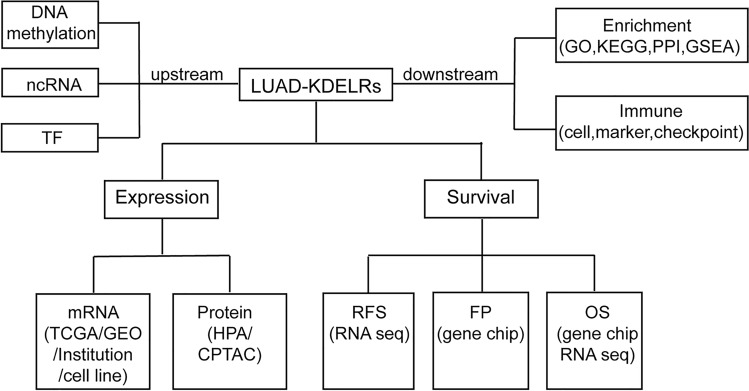
Flowchart of construction and analysis.

To explore the potential biological role of the KDELR family in LUAD, our study analyzed the characteristics of its members in LUAD from multiple perspectives, including the differential expression of mRNA and proteins, alterations of base sequences of the genes themselves (Amplification, Deletion, and Mutation), and survival analyses of related indicators. Subsequently, to explore the upstream regulatory mechanisms underlying aberrant mRNA expression, we investigated two epigenetic modification modalities (DNA methylation and non-coding RNA regulation), as well as the impact of transcription factors (TFs) on gene expression. Through enrichment analysis, we then pinpointed potential downstream pathways implicated in the dysregulated expression of KDELRs, focusing particularly on those implicated in LUAD pathogenesis. Finally, we explored the potential role of KDELRs in the tumor immune microenvironment (TIME) of LUAD by assessing their correlation with immune cells and markers. The analysis process of our study is shown in Fig. 1.

## Results

### Aberrant expression of different KDELR family members in LUAD patients

**Figure 2:**
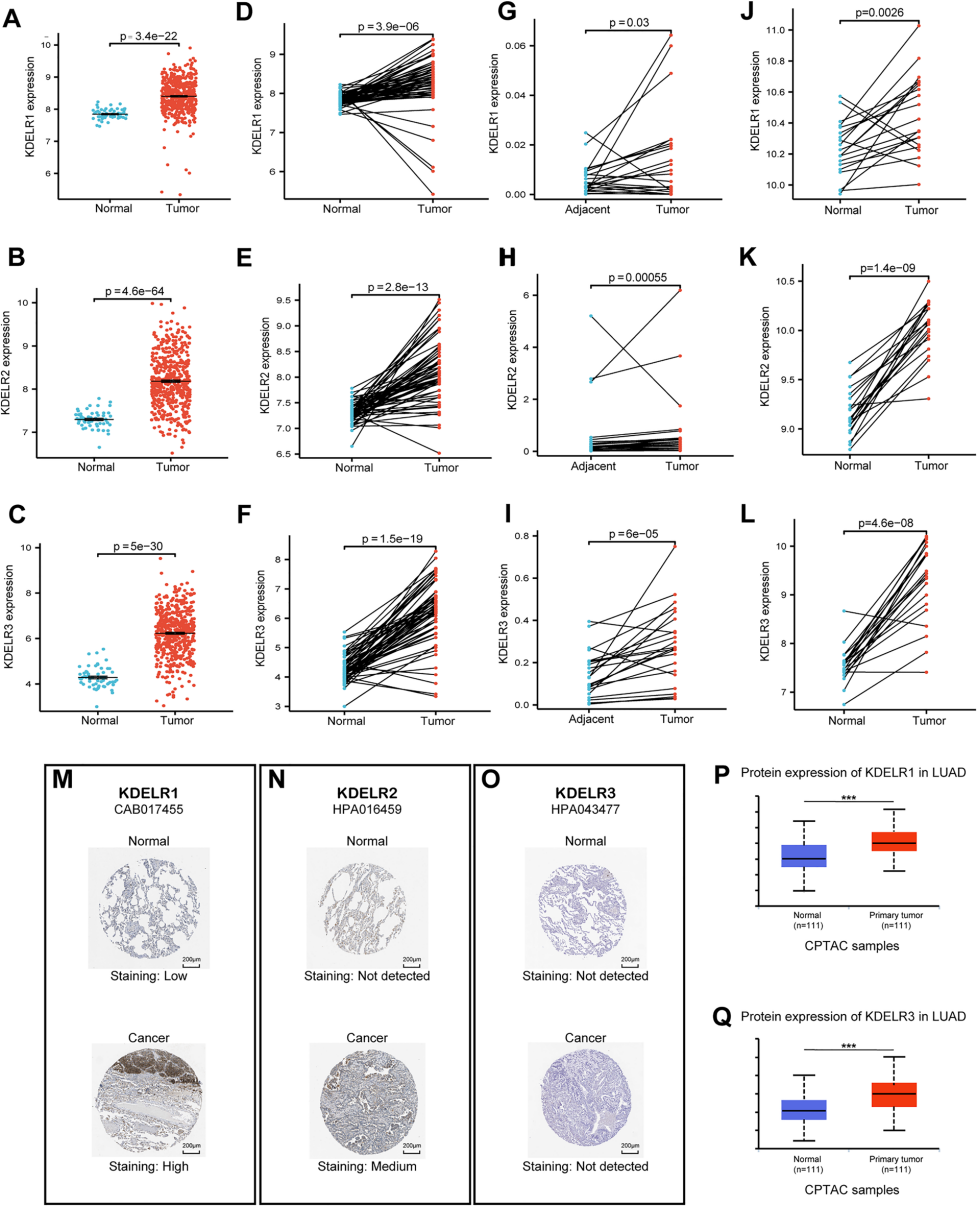
Aberrant expression of KDELR1, KDELR2, and KDELR3 in LUAD patients. (**A**–**C**) Relative mRNA expression levels of KDELR1 (**A**), KDELR2 (**B**), and KDELR3 (**C**) in LUAD tissues compared to normal tissues. (**D**–**F**) Expression of KDELR1 (**D**), KDELR2 (**E**), and KDELR3 (**F**) in 57 pairs of LUAD and adjacent tissues from TCGA. (**G**–**I**) qRT-PCR validation of KDELR1 (**G**), KDELR2 (**H**), and KDELR3 (**I**) expression in 23 pairs of clinical LUAD and adjacent tissues. (**J**–**L**) Expression of KDELR1 (**J**), KDELR2 (**K**), and KDELR3 (**L**) in 20 pairs of LUAD and adjacent tissues from GSE33532. (**M**–**O**) Representative immunohistochemical staining of KDELR1 (**M**), KDELR2 (**N**), and KDELR3 (**O**) in LUAD tissues and normal lung tissues. (**P**, **Q**) Protein expression levels of KDELR1 (**P**) and KDELR3 (**Q**) in LUAD, and normal lung tissues. (*p < 0.05, **p < 0.01 and ***p < 0.001).

After collation, we procured expression data for KDELR1, KDELR2, and KDELR3 in 513 LUAD tumor samples and 58 normal samples within The Cancer Genome Atlas (TCGA) dataset. Considering the specific characteristics and distributional properties of the data, we opted to employ either the Wilcoxon rank-sum test or the Welch’s t-test for comparative analyses. Results revealed significantly elevated mRNA expression levels of the KDELRs in LUAD tissues relative to normal tissues (p < 0.001) (Fig. 2A–C). Subsequently, we identified 57 pairs of LUAD tumors and their matched normal counterparts within the TCGA dataset. Comparative analysis of these paired samples demonstrated a significantly higher mRNA expression of all three KDELR family members in LUAD tissues compared to their corresponding normal tissues (p < 0.001) (Fig. 2D–F).

This pattern was also reproduced in an independent validation set consisting of 23 pairs of clinically matched samples from our institution, where mRNA expression levels in LUAD tissues were consistently higher than those in adjacent non-tumor tissues (Fig. 2G–I). To further validate the expression profiles of the KDELRs, we used the same method to compare the independent microarray dataset GSE33532, which consists of the expression data of 20 pairs of cancer and adjacent normal tissues. Similar results were obtained (Fig. 2J–L). For the comparison of paired samples, the Wilcoxon signed-rank test or paired-samples t-test was utilized as appropriate, depending on the data format characteristics of each group.

Next, the Human Protein Atlas (HPA) was used to directly contrast the protein expression of KDELR1/2/3 in LUAD and normal lung tissues via immunohistochemical staining. KDELR1 displayed heightened protein expression in LUAD tissues relative to its low expression in normal lung tissues (Fig. 2M). KDELR2 protein was moderately expressed in LUAD tissues but was virtually absent in normal lung tissues (Fig. 2N). Conversely, KDELR3 protein expression was undetectable in both normal and LUAD tissues (Fig. 2O). To further investigate the protein expression pattern of KDELR1/2/3, we utilized UALCAN, accessing data from the Clinical Proteomic Tumor Analysis Consortium (CPTAC) database. Despite KDELR2 being absent in the LUAD dataset, we found that total protein expression of KDELR1 and KDELR3 was significantly higher in LUAD tissues compared to normal tissues (Fig. 2P,Q. p < 0.001).

In summary, our comprehensive analysis demonstrated that both mRNA transcripts and proteins of KDELRs exhibit increased expression in LUAD patients relative to normal controls.

### Prognostic value of mRNA expression of KDELRs in LUAD patients

**Figure 3:**
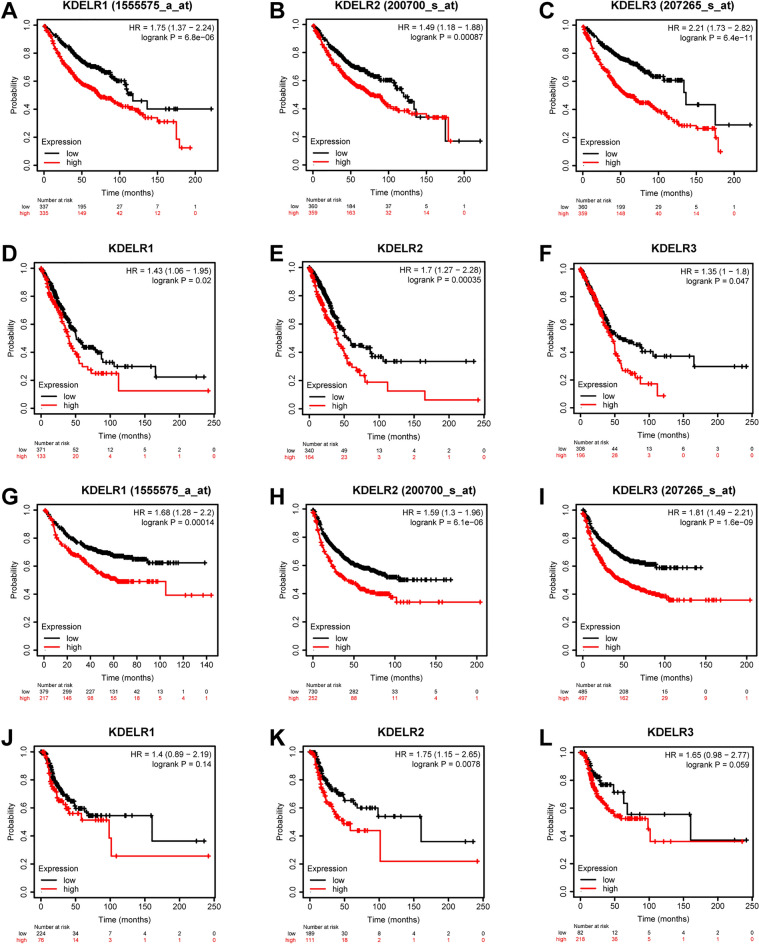
Prognostic value of mRNA expression of KDELRs in LUAD. (**A**–**C**) OS based on “gene chip” database. (**D**–**F**) OS based on “RNA seq” database. (**G**–**I**) FP based on “gene chip” database. (**J**–**L**) RFS based on “RNA seq” database.

We analyzed the correlation between KDELRs mRNA expression and multiple prognostic indicators in LUAD patients using the Kaplan Meier (K–M) plotter. As shown in Fig. 3A–C, the data from the “gene chip” database indicated that higher mRNA expression of KDELR1 (HR 1.75, 95% CI 1.37–2.24, and p = 6.8e−06), KDELR2 (HR 1.49, 95% CI 1.18–1.88, and p = 8.7E−04), and KDELR3 (HR 2.21, 95% CI 1.73–2.82, and p = 6.4E−11) were markedly related to shorter overall survival (OS) in LUAD. Analyses from the “RNA-seq” database also showed similar significant results for KDELR1 (HR 1.43, 95% CI 1.06–1.95, and p = 0.02), KDELR2 (HR 1.7, 95% CI 1.27–2.28, and p = 0.00035), KDELR3 (HR 1.35, 95% CI 1–1.8, and p = 0.047) (Fig. 3D–F). Further, we used the data from the two databases to evaluate relapse-free survival (RFS) and the first progression survival (FP). The results indicated prominent correlations between shorter FP and higher mRNA expression of KDELR1 (HR 1.68, 95% CI 1.28–2.2, and p = 0.00014), KDELR2 (HR 1.59, 95% CI 1.3–1.96, and p = 6.1E−06), KDELR3 (HR 1.81, 95% CI 1.49–2.21, and p = 1.6E−09) (Fig. 3G–I). It also showed a prominent association between shorter RFS and higher KDELR2 mRNA expression (HR 1.75, 95% CI 1.15–2.65, p = 0.0078), whereas there was also a trend towards shorter RFS with high expression of KDELR1 and KDELR3 (Fig. 3J–L). These findings collectively suggested that mRNA expression of KDELRs was closely correlated with the prognosis of LUAD and might be used as effective biomarkers for predicting the survival of LUAD patients.

### Mutations and prognosis of KDELR family genes

We analyzed genomic alterations of the KDELR family in 3513 LUAD patients across ten studies and assessed their associations with OS and disease-free survival (DFS) using the cBioPortal website. The mutation rates for KDELR1, KDELR2, and KDELR3 were found to be 0.9%, 3%, and 1.2%, respectively (Supplementary Fig. S1A). Furthermore, we found genetic alterations in KDELRs were significantly correlated with shorter OS (Supplementary Fig. S1B, p = 0.0154) and DFS (Supplementary Fig. S1C, p = 0.0126) in LUAD according to the results of K-M plots and log-rank test. These findings indicated that genetic alterations in KDELRs might have prognostic implications for LUAD patients.

### Methylation associated with KDELR expression and prognosis

**Figure 4:**
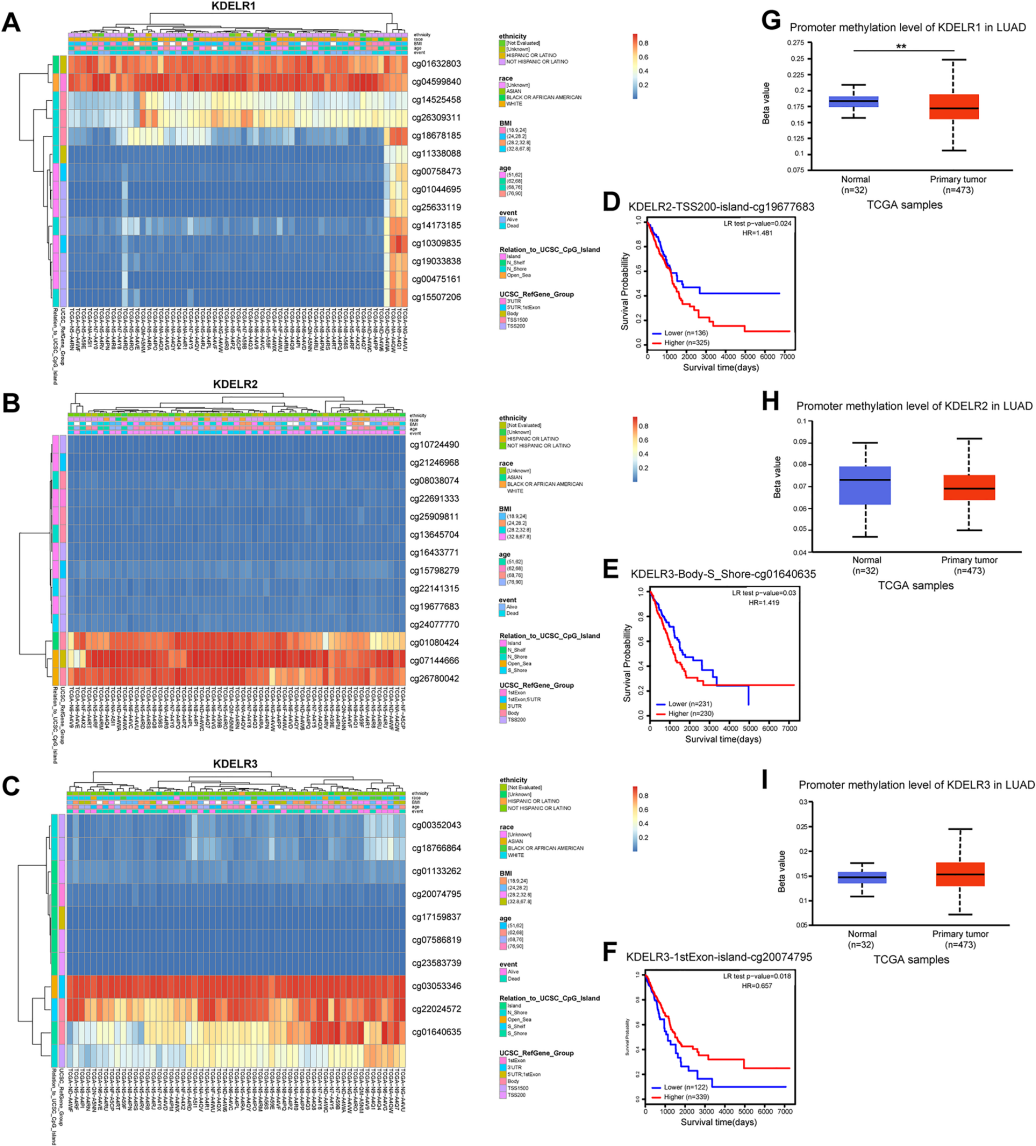
Methylation patterns of KDELR1, KDELR2, and KDELR3 in LUAD. (**A**–**C**) Visualization of methylation status of individual CpGs within the regions of KDELR1 (**A**), KDELR2 (**B**), and KDELR3 (**C**) in LUAD samples. (**D**) Kaplan–Meier survival curve for promoter methylation of KDELR2. (**E**, **F**) Kaplan–Meier survival curves for promoter methylations of KDELR3. (**G**–**I**) Promoter methylations of KDELR1 (**G**), KDELR2 (**H**), and KDELR3 (**I**) in tumor and normal tissues. (*p < 0.05, **p < 0.01 and ***p < 0.001).

As depicted in Fig. 4A–C, MethSurv was used to show the visualization of the methylation levels of individual CpGs corresponding to KDELR1, KDELR2, and KDELR3 in LUAD. Differential methylation patterns were observed across the CpG sites, potentially reflecting the complexity of epigenetic regulation of KDELR gene expression in LUAD. In the survival analysis, we discovered that LUAD patients exhibiting hypomethylation at cg19677683 within KDELR2 and at cg01640635 within KDELR3, demonstrated significantly improved OS (p < 0.05; Fig. 4D,E), Conversely, LUAD patients with hypomethylation at cg20074795 within KDELR3 had significantly worse OS (p < 0.05; Fig. 4F). In the comparative assessment utilizing UALCAN, while no significant differences were observed in the promoter methylation levels of KDELR2 and KDELR3 between TCGA-LUAD tumor tissues and their corresponding normal counterparts (Fig. 4H,I), a striking disparity was evident for KDELR1, where its promoter methylation was notably lower in tumor tissues compared to adjacent normal tissues (p < 0.01) (Fig. 4G). This finding contrasted with the mRNA expression pattern of KDELR1.

### Construction of networks of mRNA–miRNA–lncRNA

**Figure 5:**
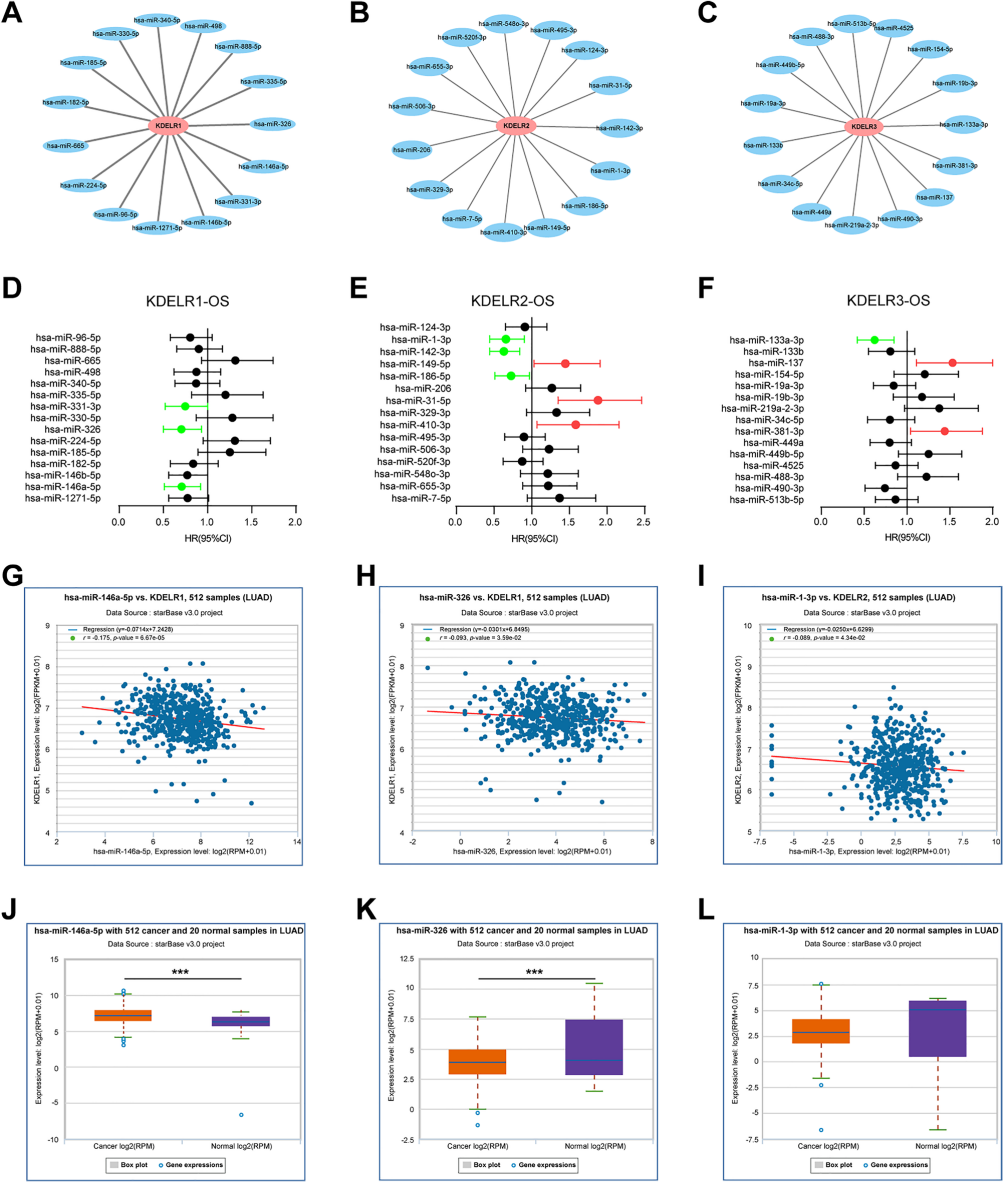
Identification of upstream miRNAs regulating KDELR1, KDELR2, and KDELR3 in LUAD. (**A**–**C**) miRNA-KDELR1/2/3 network. (**D**–**F**) Prognostic values of potential upstream miRNAs of KDELR1/2/3 in LUAD. High expression of miRNAs was significantly correlated with favorable OS (green) or unfavorable OS (red). The p-value was set at 0.05. (**G**, **H**) Correlation analysis of hsa-miR-146a-5p (**G**) and hsa-miR-326 (**H**) with KDELR1 expression in LUAD. (**I**) Correlation analysis of hsa-miR-1-3p with KDELR2 expression in LUAD. (**J**–**L**) Expression level of hsa-miR-146a-5p, hsa-miR-326, and hsa-miR-1-3p in LUAD (red) versus normal lung tissues (blue) (*p < 0.05, **p < 0.01 and ***p < 0.001).

Employing the comprehensive miRNA prediction capabilities of the seven integrated programs within ENCORI, we identified15 upstream miRNAs with the highest stringency targeting KDELR1/2/3 under the “CLIP Data” (Supplementary Table S1). For better visualization, miRNA-KDELR1/2/3 subnetworks were established using Cytoscape software (Fig. 5A–C). Subsequently, the K–M plotter was used to evaluate the prognostic significance of these miRNAs in LUAD. Among all miRNAs predicted for KDELR1, elevated expression of hsa-miR-331-3p, hsa-miR-146a-5p, and hsa-miR-326 was related to improved OS in LUAD (Fig. 5D). For KDELR2, upregulation of hsa-miR-142-3p, hsa-miR-186-5p, and hsa-miR-1-3p correlated with favorable prognosis, whereas hsa-miR-410-3p, hsa-miR-149-5p, and hsa-miR-31-5p indicated poor prognosis (Fig. 5E). In the case of KDELR3, higher expression of has-miR-133a-3p showed favorable OS, but higher expression of hsa-miR-137 and hsa-miR-381-3p was associated with unfavorable OS (Fig. 5F). Given the functional mechanism of miRNAs and the oncogenic effects of KDELR1/2/3, the upstream miRNAs of the three genes should exert inhibitory effects in LUAD. Consequently, seven miRNA-mRNA pairs, including hsa-miR-331-3p, hsa-miR-146a-5p, hsa-miR-326, hsa-miR-142-3p, hsa-miR-186-5p, hsa-miR-1-3p and hsa-miR-133a-3p were selected for further expression correlation analysis. Based on the regulatory mechanism of miRNAs suppressing target gene expression, we inferred that there should exist a negative association between KDELR family genes and the miRNAs. Hence, we performed expression correlation analysis using ENCORI. Three of the seven miRNA-mRNA pairs exhibited significant negative correlations KDELR family members. Specifically, hsa-miR-146a-5p and has-miR-326 were negatively correlated with KDELR1, and hsa-miR-1-3p was negatively related to KDELR2 in LUAD (Fig. 5G–I). The correlations between KDELRs and the remaining four miRNAs were not statistically significant. As presented in Fig. 5J–L, has-miR-326 was markedly downregulated (p < 0.001), while has-miR-146a-5p was markedly upregulated in LUAD (p < 0.001). Collectively, the hsa-miR-326-KDELR1 axis represents a promising regulatory pathway involved in LUAD tumor progression.

**Figure 6:**
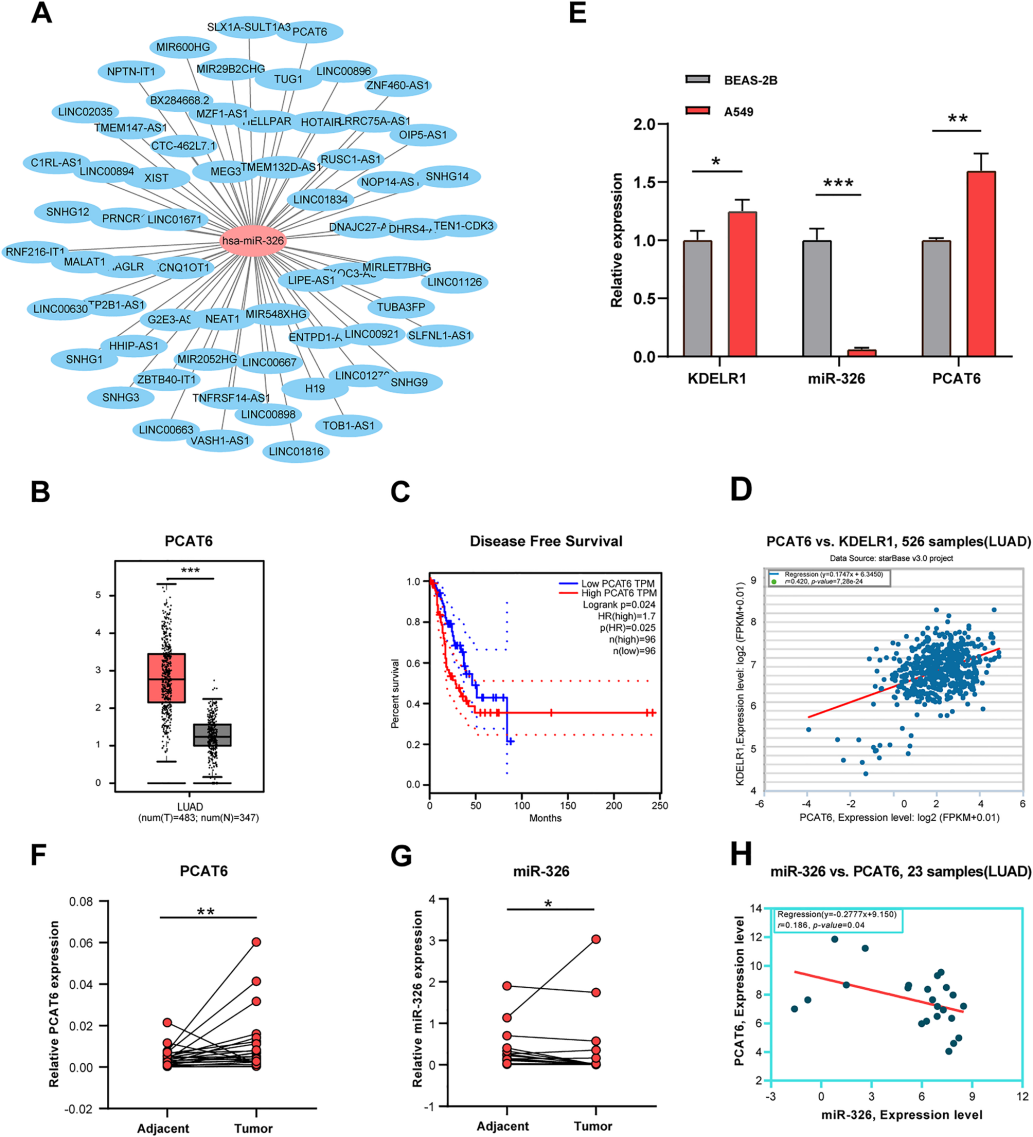
Identification of upstream lncRNAs regulator of hsa-miR-326 in LUAD. (**A**) Potential lncRNAs regulating hsa-miR-326. (**B**) Expression levels of PCAT6 in LUAD (red), and normal lung tissues (gray). (**C**) Prognostic value of PCAT6 in LUAD. (**D**) Correlation analysis of PCAT6 and KDELR1 expression in LUAD based on ENCORI database. (**E**) Quantification of relative expression levels of KDELR1, miR-326, and PCAT6 in cell lines using qRT-PCR. (**F**–**G**) qRT-PCR validation of PCAT6 and miR-326 expression in 23 pairs of LUAD tissues and adjacent tissues. (**H**) Correlation analysis of miR-326 and PCTA6 in 23 pairs of LUAD tissues. (*p < 0.05, **p < 0.01 and ***p < 0.001).

Subsequently, we utilized the ENCORI data platform to predict hsa-miR-326 upstream-interacting lncRNAs. In total, 64 potential lncRNAs were predicted. We constructed lncRNA-hsa-miR-326 regulatory networks using Cytoscape software to enhance visualization (Fig. 6A). Then, GEPIA2 was utilized to identify the expression levels of these candidate lncRNAs in LUAD. As depicted in Fig. 6B, among the 64 lncRNAs, only PCAT6 was significantly upregulated compared to normal controls in LUAD (p < 0.001). GEPIA2 was then used to evaluate the prognostic relevance of PCAT6 in LUAD. Elevated PCAT6 expression was found to be associated with shorter DFS in LUAD (p < 0.05) (Fig. 6C). According to the competing endogenous RNA (ceRNA) hypothesis^18^, ceRNAs can competitively bind to shared miRNAs, thereby indirectly affecting each other’s expression. Thus, lncRNAs and their related miRNAs should exhibit a negative association, whereas lncRNAs and mRNAs should show a positive association. As shown in Fig. 6D, PCAT6 was indeed found to be significantly positively associated with KDELR1 in LUAD based on the ENCORI database. Considering expression, survival, and correlation analyses, we concluded that PCAT6 might be the most potential upstream lncRNA in the hsa-miR-326/KDELR1 axis in LUAD.

Experimental validation by Quantitative reverse transcription polymerase chain reaction (qRT-PCR) in clinical tissue and cell line samples revealed that KDELR1 and PCAT6 had higher expression, whereas hsa-miR-326 showed lower expression in the A549 cell line compared to the BEAS-2B cell line (Fig. 6E). Analogous results were obtained when comparing 23 clinical LUAD tissues and adjacent matched normal tissues (Figs. 1D, 6F,G). As shown in Fig. 6H, PCAT6 was significantly negatively associated with has-miR-326 in clinical LUAD tissues. These validation results largely concurred with the outcomes of our earlier database analyses.

### Construction of transcription factor regulatory networks

Based on comparative analysis of the promoter sequences of the target genes with respect to the DNA-binding domains of TFs, followed by stringent filtration criteria encompassing "forward strands" and "score > 20", the estimated count of TFs potentially interacting with KDELR1, KDELR2, and KDELR3 was respectively culled down to 14, 43, and 23. Subsequent Venn diagram intersection (Supplementary Fig. S2A) led to the selection of three key TFs—SPI1, EP300, and MAZ. Bioinformatic analyses (Supplementary Fig. S2B) uncovered the most highly predicted binding elements for these three key TFs within the corresponding promoter regions of KDELR1/2/3.

Previously, using tools like ENCORI and K-M plotter, seven upstream miRNAs belonging to the KDELRs family, including has-miR-331-3p, hsa-miR-146a-5p, hsa-miR-326, hsa-miR-142-3p, hsamiR-186-5p, hsa-miR-1-3p and hsa-miR-133a-3p, were selected to be tumor suppressive miRNAs. Through the "enrichment analysis" program of the TransmiR v2.0 database, we uncovered 214 TFs associated with these seven miRNAs. Proceeding with the condition of "P < 0.05" and "count > 1", we then selected 11 TFs linked to 5 miRNAs. The relationship between these TFs and their regulatory network was depicted in Supplementary Fig. S2C. We consequently constructed a regulatory network comprising TFs-miRNAs-target genes, including 11 TFs, 5 miRNAs, and 3 KDELRs family genes. Examples included the regulatory pathways RUNX1T1-miR326-KDELR1, CHD1-miR142-KDELR2, TNFSF12-miR133a-KDELR3.

### Functional enrichment analysis and PPI networks of KDELRs

The functions of KDELR1/2/3 and a cohort of 60 genes (selected from GEPIA2) sharing similar expression patterns were inferred via Gene Ontology (GO) and Kyoto Encyclopedia of Genes and Genomes (KEGG) pathway analysis executed in Metascape^19–21^. These annotations were classified into four main categories: GO biological process group (10 items), GO cellular component group (7 items), GO molecular function group (2 items), and KEGG pathway group (1 item). KDLR1/2/3 and the comparable 60 genes were predominantly enriched in vesicle-mediated transport processes from the ER to the Golgi apparatus, as evidenced in Supplementary Fig. S3A.

Considering that the molecular machinery and metabolism requirements of malignant tumors necessitate functional interactions among proteins, we proceeded to analyze the protein–protein interaction (PPI) network of KDELRs and the proteins encoded by the 60 similarly expressed genes (Supplementary Fig. S3B). From this PPI network encompassing 63 proteins, the two most significant Molecular Complex Detection (MCODE) components were extracted. Their biological functions were primarily related to Golgi vesicle transport, retrograde vesicle-mediated transport from Golgi to ER, ER to Golgi vesicle-mediated transport, and the Sphingolipid signaling pathway (Supplementary Fig. S3C). Furthermore, we procured PPI network data for KDELR1/2/3 proteins, listing the top 10 predicted functional interactors in Supplementary Fig. S3D, including ARFGAP1, ARF1, COPB1, ARFGAP3, COPA, ASAP1, CLTA, XBP1, ASAP2, ARAP2.

**Figure 7:**
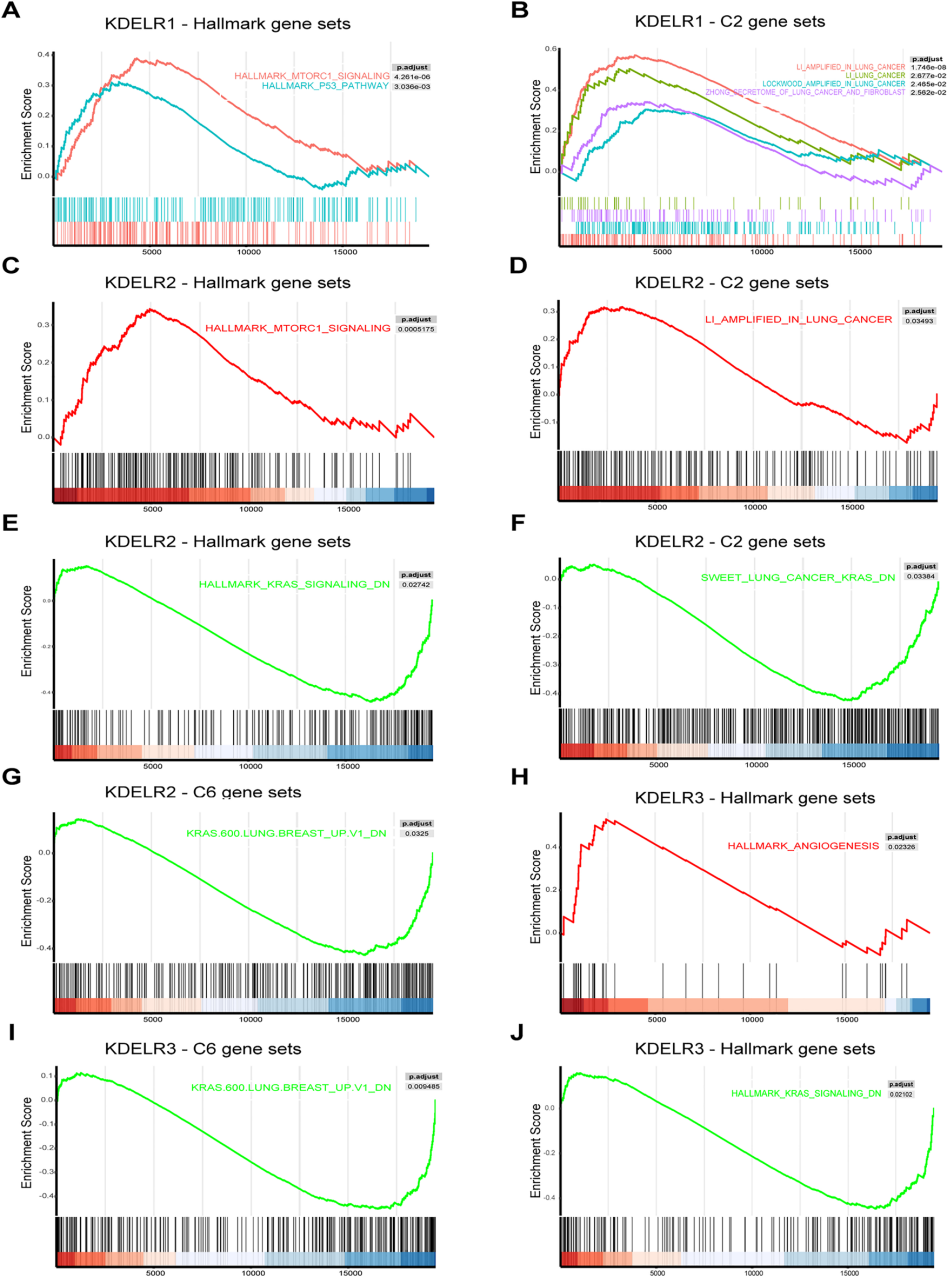
GSEA for samples with high and low KDELR expression. (**A**, **B**) Enriched gene sets in HALLMARK and C2 collections associated with high KDELR1 expression. (**C**, **D**) Enriched gene sets in HALLMARK and C2 collections associated with high KDELR2 expression. (**E**–**G**) Enriched gene sets in HALLMARK, C2, and C6 collections associated with low KDELR2 expression. (**H**) Enriched gene sets in the HALLMARK collection associated with high KDELR3 expression. (**I**–**J**) Enriched gene sets in C6 and HALLMARK collections associated with low KDELR3 expression.

Dividing the samples based on median expression levels, we categorized KDELR1/2/3 into high and low expression groups and subsequently analyzed the differential expression of 19,934 genes across these groups in TCGA dataset, ranking them according to their degree of upregulation. By contrasting the enrichment significance of target gene sets in the Molecular Signatures Database v7.0 against this ranked list, we conducted Gene Set Enrichment Analysis (GSEA). GSEA results were obtained by comparing the enrichment significance of the target gene sets in Molecular Signatures Databases v7.0 in this sort. The GSEA results indicated that the genes in the KDELR1 high-expression group were significantly enriched in the MTORC1_SIGNALING and P53_PATHWAY within the HALLMARK collection (Fig. 7A) and concurrently enriched in four gene sets within the C2 collection, all of which were overexpressed in multiple lung cancer cell lines (Fig. 7B). The genes in KDELR2 high-expression group were enriched in the MTORC1_SIGNALING in HALLMARK collection (Fig. 7C) and one gene set in the C2 collection that is upregulated in LUAD cell lines (Fig. 7D). The genes in the KDELR2 low-expression group were enriched in gene sets representing downregulated genes in KRAS-mutated lung cancer cell lines and animal models, sourced from HALLMARK, C2, and C6 collections (Fig. 7E–G). Similarly, the KDELR3 high-expression group was enriched in the ANGIOGENESIS pathway within the HALLMARK collection (Fig. 7H), whereas the KDELR3 low-expression group was enriched in gene sets reflecting downregulated genes in KRAS-mutated lung cancer cell lines. Like KDELR2, these 2 gene sets are derived from the C6 and Hallmark collections, respectively (Fig. 7I,J).

### Correlation of KDELR expression with immune characteristics

**Figure 8:**
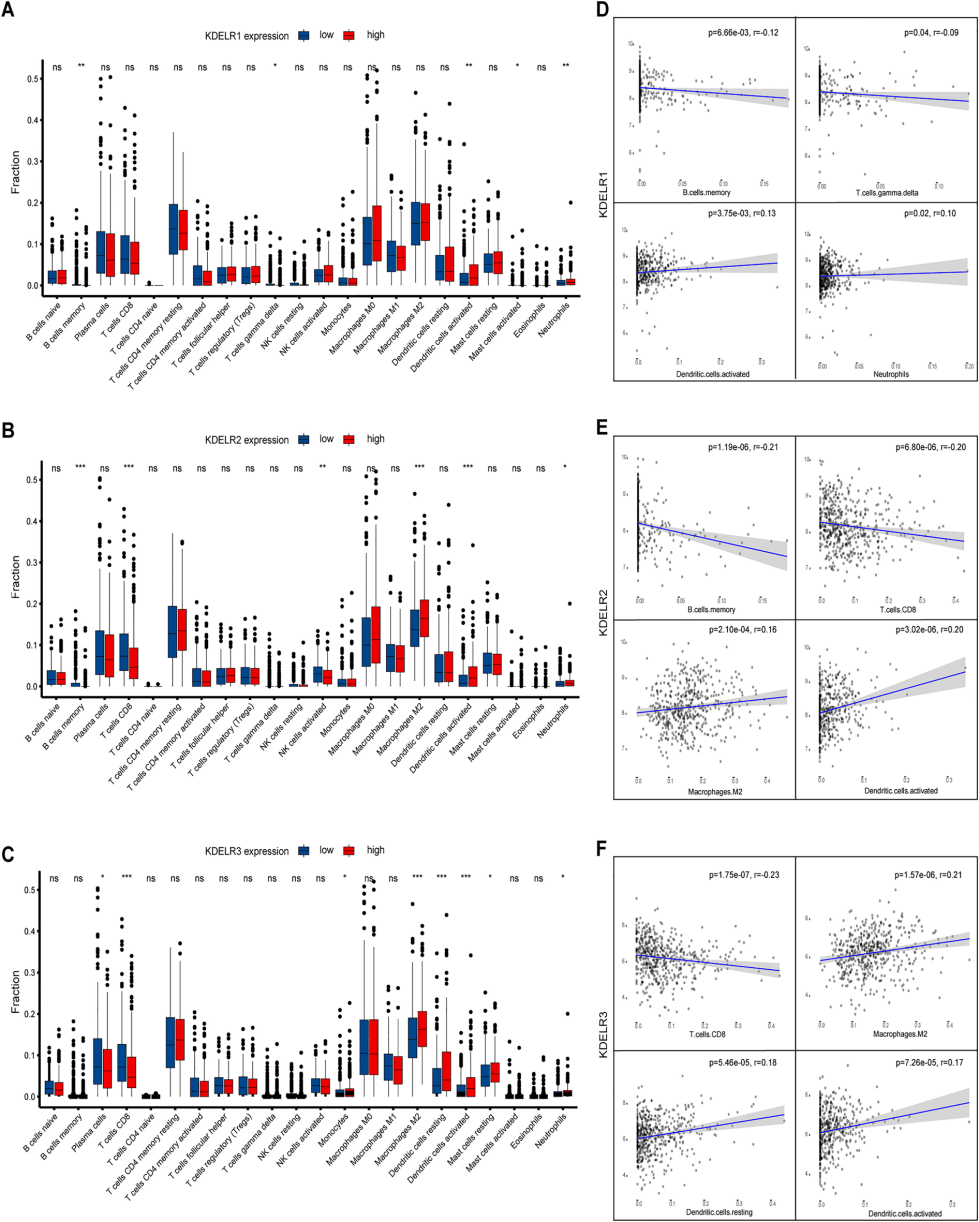
Correlation of TICs proportions with KDELR1, KDELR2, and KDELR3 expression. (**A**–**C**) Differential ratios of 22 immune cell types between LUAD tumor samples exhibiting high versus low expression of KDELR1 (**A**), KDELR2 (**B**), and KDELR3 (**C**). The Wilcoxon rank-sum test was applied to determine statistical significance (*p < 0.05, **p < 0.01 and ***p < 0.001). (**D**–**F**) Correlation of the top 4 TIC proportions with KDELR1 (**D**), KDELR2 (**E**), and KDELR3 (**F**) expression (p < 0.05). Pearson's correlation coefficient was utilized for the correlation analysis.

Among the patients with cancer, the frequency of lymphocytes infiltrating the tumor independently predicts survival and lymph node metastasis^22–24^. Thus, we stratified LUAD samples from the TCGA database into high- and low-expression groups based on the median expression levels of KDELR1/2/3, respectively, and subsequently assessed the proportion differences of the 22 tumor-infiltrating immune cells (TICs) between these groups. It was observed that, correspondingly, six, eight, and nine immune cell populations displayed significantly distinct proportions in the high versus low expression groups of KDELR1, KDELR2, and KDELR3, respectively (Fig. 8A–C, p < 0.05).

Subsequently, we examined the correlation between the expression levels of KDELR1/2/3 and the proportions of the 22 immune cell types in all LUAD samples. This analysis revealed significant correlations between five, six, and eight immune cell types with KDELR1, KDELR2, and KDELR3, respectively. Upon taking the intersection of the results from these two analytical approaches, the top four immune cell types most strongly associated with KDELR1 expression were B cells memory, T cells gamma delta, Dendritic cells activated, and Neutrophils (Fig. 8D). Similarly, for KDELR2, the top four significantly correlated immune cells were B cells memory, T cells CD8, Macrophages M2, and Dendritic cells activated (Fig. 8E). Whereas for KDELR3, they were T cells CD8, Macrophages M2, Dendritic cells resting, and Dendritic cells activated (Fig. 8F). These findings further substantiated that the levels of KDELRs exerted a significant influence on the immunological activity within the tumor microenvironment (TME).

To further elucidate the potential role of KDELR family genes in the infiltration of diverse immunocytes in LUAD patients, the TIMER database was utilized to investigate connections between KDELR family genes and a set of immunological markers widely recognized as counterparts of different immunocytes. Upon adjusting for tumor purity, we detected significant associations between the expression of KDELR family members and markers of CD8 + T cell (CD8A, CD8B), T cell (general) (CD3D, CD3E, CD2), M1 macrophage (COX2), Neutrophils (CD15, CCR7), NK cell (KIR3DL2), Th1 (T-BET), Th17 (IL17A) in LUAD (P < 0.05; Supplementary Table S2). Notably, all markers of CD8 + T cells, displayed significant negative correlations with the expression of all KDELR family members. Moreover, the proportion of CD8 + T cells demonstrated a significant negative correlation with KDELR2 and KDELR3 expression, while exhibiting a trend towards negative correlation with KDELR1 expression.

Immune checkpoints, such as programmed cell death protein 1 (PD1), programmed death-ligand 1 (PD-L1) and cytotoxic T-lymphocyte-associated protein 4 (CTLA-4), play a pivotal role in the orchestration of tumor immune escape mechanisms^25^. Given the putative oncogenic involvement of KDELRs in LUAD, we sought to investigate the possible association of KDELRs with these immunoregulatory molecules. The correlation analysis revealed a strikingly negative association between the genetic expression levels of KDELR1, KDELR2, and KDELR3 and that of CTLA-4 in LUAD. Specifically, the correlation coefficients (cor) and corresponding p-values were as follows: cor = − 0.14, p = 1.89e−03 for KDELR1 versus CTLA-4; cor = − 0.11, p = 1.46e−02 for KDELR2 versus CTLA-4; and cor = − 0.165, p = 2.28e−04 for KDELR3 versus CTLA-4.

## Discussion

Recent studies have increasingly implicated diverse molecules in the occurrence and development of lung cancer, especially LUAD^26,27^. Whereas KDELR, as a receptor for ER proteins, maintains a dynamic balance between the Golgi and the ER^4,5^, and circulates between the Golgi and the plasma membrane to contribute to the binding of ER proteins on the cell surface, thereby promoting cell proliferation and migration^10,11^. In addition to trans-activation of the epidermal growth factor receptor-STAT3 signaling pathway^12^, other signaling networks downstream of its own are unclear. Therefore, in this paper, we synthesized data from various aspects in the expectation of finding the potential biological significance of KDLER family genes in lung adenocarcinoma.

The KDELR family, comprising KDELR1 (chr19q13.33), KDELR2 (chr7p22.1), and KDELR3 (chr22q13.1), plays pivotal roles in various cancers^13–17^. Our study discovered higher mRNA and protein expression of KDELR family members in LUAD tissues relative to normal counterparts. Furthermore, the heightened expression was prominently correlated with poorer prognostic indicators in LUAD. These results implicated KDELRs as exerting a possible potential tumorigenic effect in LUAD.

DNA methylation, a pivotal epigenetic modification, has long been studied for its role in transcriptional repression. When methylation occurs within gene promoter regions, CpG islands become methylated, preventing protein binding and leading to transcriptional silencing^28^. Conversely, low methylation levels in gene promoter regions can fail to inhibit oncogene transcription, contributing to tumor initiation. Jones et al.^29^ confirmed that methyl-CpG-binding domain (MBD) proteins initially recognized methylated CpGs in promoter regions, subsequently recruiting histone deacetylase complexes to repress downstream gene expression. Recent studies further suggested that DNA methylation had a critical effect on the regulation of gene expression^30–33^. We found that KDELR1 promoter methylation in LUAD cancer tissues was prominently lower than that in neighboring normal tissues, contrasting with the gene expression of KDELR1. This suggests that reduced promoter methylation may contribute to the upregulated expression of KDELR1 in LUAD samples.

miRNAs are a group of ncRNA molecules that have been established as important regulators of gene expression^34–36^. Following survival, correlation, expression analysis, and qRT-PCR validation, we identified has-miR-326 as the most significant tumor-suppressive upstream miRNA of KDELR1. Previous studies have identified that hsa-miR-326 is involved in embryonic development, immunoregulation, tumorigenesis, tumor growth, chemoresistance, cell invasion, and apoptosis^37,38^. Based on the ceRNA hypothesis, the ceRNA molecules (mRNAs, lncRNAs, pseudogenes, etc.) were able to compete to bind the same miRNAs to regulate each’other's expression levels via the miRNA response element^18,39^. Therefore, we predicted and identified potential lncRNAs acting as upstream regulators of the hsa-miR-326/KDELR1 axis. PCAT6 was screened out following expression, survival, and correlation analyses, as well as qRT-PCR validation. Recent studies identified that PCAT6 had the function of an oncogene in various tumors. Liu et al.^40^ demonstrated that PCAT6 promotes prostate cancer development by sponging miR-326 as well as enhancing Hnrnpa2b1 expression. Another study also demonstrated that PCAT6 regulated RhoA-ROCK and miR-326 signaling pathways, driving M2 polarization of macrophages in cholangiocarcinoma^41^. Collectively, the PCAT6/hsa-miR-326/KDELR1 axis represented a putative regulatory pathway, which might be another reason for the upregulation of KDELR1 expression in LUAD samples.

TFs can potentially modulate the mRNA expression both directly, through transcriptional regulation, and indirectly, by affecting the activity of miRNAs that target the mRNA. To explore potential TFs targeting KDELRs, we aligned the sequences of their respective promoters and identified SPI1, EP300, and MAZ as critical TFs for KDELR regulation. These TFs may directly participate in the initiation of KDELR transcription, thereby influencing their mRNA expression levels. Previous research has implicated these TFs in cancer genesis, proliferation, invasion, and metastasis^42–44^. On the other hand, we employed the enrichment analysis to construct a regulatory network of 11 TFs-5 miRNAs-KDELR1/2/3 relevant to LUAD progression, encompassing. This network illustrated the interconnected regulatory relationships among TFs, miRNAs, and target genes, revealing potential cascades controlling KDELR expression in LUAD. This dual mode of action illustrated the intricate interactions among TFs, miRNAs, and KDELR mRNAs in the biological regulation of cancer.

In our study, PPI network analyses were used to predict ARFGAP1, ARF1, COPB1, ARFGAP3, and COPA as the top 5 functional interactors of KDELRs. Each of these interactors has been linked to various cancers: ARFGAP1 acts as a critical regulator of mTORC1 with potential for cancer therapeutics^45^; downregulation of ARF1 suppresses NSCLC progression^46^; COPB1 emerges as a significant prognostic biomarker for pan-cancer^47^; as a target gene for androgens, ARFGAP3 promotes prostate cancer progression and metastasis^48^; and COPA is recognized as a prognostic biomarker and drug target in cervical cancer^49^. Additionally, analysis of KDELRs by GSEA revealed gene sets that enriched in the high-expression KDELRs group encompassed "MTORC1_SIGNALING", "P53_PATHWAY", and "ANGIOGENESIS" from the "HALLMARK collection". These hallmarks represent upregulated gene sets involved in cell growth, tumorigenesis, and angiogenesis, respectively^50^. Furthermore, the high-expression KDELRs group was enriched for four gene sets from the "C2 collection" that display elevated expression across multiple lung cancer cell lines^51–53^. And the gene sets enriched in the low-expression group of KDELRs are mainly down-regulated genes in KRAS-mutated lung cancer cell lines and animal models^54^. These GSEA results collectively demonstrate a positive correlation between high KDELR1/2/3 expression and multiple upregulated gene sets in lung cancer, as well as a negative correlation with downregulated gene sets. These associations suggested that KDELR family members may be indirectly involved in these pathways leading to tumorigenesis and development.

Numerous studies have established that the immune microenvironment of tumors significantly impacts tumor progression, recurrence, and patient prognosis^55,56^. In cancer patients, the frequency of lymphocyte infiltration in tumors independently predicts survival and lymph node metastasis^22–24^ Our findings suggest that KDELR overexpression in LUAD may negatively regulate CD8 + T cell infiltration. CD8 + cytotoxic T lymphocytes are considered the primary immune cells for targeted cancer therapies, which kill infected or tumor cells by secreting large amounts of IFN-γ and protease granzyme B^22,57^. Thus, elevated KDELR expression in LUAD may result in reduced CD8 + T cell presence within the TIME, diminishing the host's capacity to suppress tumor growth. Moreover, for immunotherapy to be effective, adequate expression of immune checkpoints, such as PD1/PD-L1 or CTLA-4, which play a pivotal role in tumor immune escape, is required^25^. We found a potentially antagonistic relationship between KDELRs and CTLA-4 in the context of LUAD, which may hold implications for unraveling the complex immunological landscape of this malignancy and guiding the development of innovative therapeutic strategies targeting both KDELRs and immune checkpoint axes. In summary, the KDELR family appears to influence the efficacy of immunotherapy in LUAD, offering valuable insights for future investigations.

This study employs the bioinformatic approach, conducting comprehensive, multi-omics analyses to elucidate the potential roles of the KDELR family genes in lung adenocarcinoma, thereby augmenting and refining the exploration of KDELR functionalities within the realm of oncology. The investigation encompasses a broad spectrum of data, including datasets from TCGA, GEO, and independent clinical cohorts from our institution, ensuring robust validation across diverse sources. Upon comparing various types of data, KDELR1 emerged as the member with the most discernible indicators. Consequently, we used KDELR1 as an example to summarize the upstream regulatory mechanisms and downstream pathways associated with its abnormal expression, as depicted in Supplementary Fig. S4. The upstream part includes a competing endogenous RNA (ceRNA) network comprising PCAT6, hsa-miR-326, and KDELR1. It also contains the promoter hypomethylation and three key TFs—SPI1, EP300, and MAZ—alongside a broader TFs-miRNAs-target genes network encompassing 4 TFs, was implicated in modulating the dysregulation of KDELR1 expression in LUAD. The downstream part includes MTORC1_SIGNALING and P53_PATHWAY, two key pathways for tumorigenesis and disease progression obtained from GSEA analysis. KDELR1 might play an indirect role in coordinating these two oncogenic pathways. It also includes B cell memory, T cell γ-δ, and T cell CD8 + in the immune microenvironment, and overexpression of KDELR1 in LUAD negatively regulated their infiltration, which might result in an immunosuppressive environment that favored tumor growth.

Our study has several limitations. On one hand, the specific functions of KDELRs in LUAD needs further experimental validation. With this in mind, we have outlined plans for upcoming experiments, including designing and constructing shRNA expression vectors targeting the KDELR family genes, leveraging lentiviral packaging systems to establish stable LUAD cell lines with knocked-down KDELR expression. These cell lines will then serve as models for comparative assays evaluating cellular functions such as proliferation, invasion, migration, cell cycle, and apoptosis. On the other hand, the potential oncogenic mechanisms involving KDELRs in LUAD require substantiation through additional experimentation, particularly focusing on KDELR’s impact on LUAD-associated signaling pathways and immune activities. To elucidate these mechanisms, we intend to follow functional assays with assessments of changes in the expression of signaling pathway marker molecules before and after target gene knockdown in our cell line models.

To conclude, the current study suggested that the KDELRs are critical signaling molecules that are highly expressed in LUAD and positively associated with poor prognosis. Methylation, ceRNA networks, and TFs are identified as potential causes of aberrant KDELR mRNA expression in LUAD. Furthermore, KDELR family members may indirectly affect pathways driving tumorigenesis and progression. Elevated KDELR expression in LUAD is associated with reduced CD8 + T cell presence within the TIME, compromising the host's ability to suppress tumor growth. Overall, these findings may provide guidance for the development of prognostic markers and novel therapeutic strategies for LUAD.

## Materials and methods

### Gene expression analysis

Utilizing the R package "TCGAbiolinks," we retrieved relevant data for LUAD from The Cancer Genome Atlas (TCGA) database. Following rigorous data preprocessing steps, including deduplication and handling of missing values, we obtained Transcriptome RNA-seq data for 571 samples along with their corresponding clinical information. Among these data, we extracted expression levels of KDELR1, KDELR2, and KDELR3 in both tumor and normal tissue samples. In addition to the TCGA-based analyses, we validated our findings using an independent dataset, GSE33532, which was downloaded from the Gene Expression Omnibus (GEO) database and a group of clinical specimens from the First Hospital of Jilin University.

Considering the specific characteristics and distributional properties of the obtained expression data, appropriate statistical methods were selected to perform the analyses. Subsequently, we employed the R package "ggplot2" for data visualization, generating scatter plots and paired plots to graphically depict the expression patterns of KDELRs across different sample groups.

### Protein expression analysis

We employed two distinct databases to examine protein expression profiles: the Human Protein Atlas (HPA), based on immunohistochemical techniques, and the Clinical Proteomic Tumor Analysis Consortium (CPTAC) database, utilizing mass spectrometry methodologies. The HPA website (https://www.proteinatlas.org) houses protein expression data derived from immunohistochemistry on 44 diverse normal tissues and 20 prevalent cancer types^58^. UALCAN (http://ualcan.path.uab.edu), on the other hand, was an analytical tool grounded in the combined datasets of CPTAC and TCGA^59,60^. In the present study, these resources were leveraged to elucidate differences in the protein expression levels of KDELR1, KDELR2, and KDELR3 between LUAD and normal lung tissues.

### Survival analysis

We analyzed the correlation between KDELRs mRNA expression and multiple prognostic indicators in LUAD patients using the Kaplan Meier (K–M) plotter (http://kmplot.com/analysis/)^61^. The platform integrates data from the Gene Expression Omnibus (GEO), European Genome-phenome Archive (EGA), and TCGA databases, utilizing gene expression data generated via a combination of microarray and sequencing technologies. This comprehensive resource enables the evaluation of associations between the expression of all genes and patient survival across more than 30,000 samples from 21 distinct malignancies. It can also be used to assess the prognostic value of mRNAs and microRNAs in tumors^62,63^.

### Amplification, deletion and mutation analysis

We analyzed the genetic alteration of KDELRs using the cBioPortal (www.cbioportal.org), incorporating data from ten major cancer research initiatives, including TCGA. This site provides visual and multidimensional cancer genomics data and the association between mutations and survival^64,65^.

### Methylation analysis

MethSurv (https://biit.cs.ut.ee/methsurv/) is an online platform specifically designed for conducting survival analyses of DNA methylation biomarkers using the Cox proportional hazards model, drawing upon data from TCGA. This comprehensive dataset incorporates DNA methylation microarray data, generated using the Illumina HumanMethylation450 platform, encompassing over 10,000 samples across 34 distinct cancer types^66^. For the purposes of our investigation, we utilized MethSurv's "Gene visualization" feature to conduct cluster analysis of individual CpG sites within the target KDELR genes, presenting the results in a heatmap format. Subsequent survival analyses were then performed on these CpGs, seeking to establish correlations between methylation patterns, patient clinical characteristics, and gene subregions. Recognizing that methylation within gene promoter regions frequently functions to suppress gene transcription^28^, we further employed UALCAN (http://ualcan.path.uab.edu) to comparatively assess methylation levels in the promoter regions of KDELR genes between LUAD and normal tissues.

### Networks of mRNA–miRNA–lncRNA

In the present study, we employed a relatively exhaustive miRNA prediction approach facilitated by ENCORI (http://starbase.sysu.edu.cn/), a web-based platform dedicated to investigating RNA-miRNA-lncRNA interactions, drawing upon data from RNA-RNA interactome, CLIP-seq and degradome-seq^67,68^. Seven computational algorithms (microT, miRanda, miRmap, PicTar, PITA, RNA22, and TargetScan) were utilized to forecast potential upstream miRNAs that could interact with KDELR1, KDELR2, and KDELR3. miRNAs predicted by multiple programs were chosen for further analysis. Additionally, ENCORI was utilized to compare miRNA expression levels between LUAD and normal control samples, as well as to identify lncRNAs potentially binding to the identified miRNAs.

Subsequently, GEPIA2 (http://gepia2.cancer-pku.cn/index.html), which harnesses data from the Genotype-Tissue Expression (GTEx) project and TCGA, was used to discern expression differences of lncRNAs between LUAD and normal tissues, and to conduct survival analyses for these lncRNAs in the context of LUAD^69^. Complementarily, we employed qRT-PCR to assess the expression levels of each component on the KDELR1/hsa-miR-326/PCAT6 axis in both clinical tissues and cell lines.

### Transcription factor analysis

TF targets of the KDELRs were investigated by aligning the sequences of their promoters against the Animal Transcription Factor Database (AnimalTFDB) version 3.0 (http://bioinfo.life.hust.edu.cn/AnimalTFDB/). The AnimalTFDB3.0 is an integrated database that includes the classification and annotation of transcription cofactors, and genome-wide TFs^70^.

Subsequently, we performed TF-related enrichment analysis for the set of upstream miRNAs of KDELR1/2/3 previously identified, utilizing TransmiR version 2.0 (http://www.cuilab.cn/transmir), which is used for elucidating regulatory relationships between TFs and miRNAs^71^.

### Functional enrichment analysis and PPI networks

To elucidate the functional implications and pathways associated with KDELRs and a cohort of 60 genes (20 genes corresponding to each KDELR member) with similar expression patterns, we employed Metascape (accessible at http://metascape.org), an online platform offering a comprehensive suite for gene list annotation and analysis^72^. Statistically significant terms were determined based on a P-value cutoff of < 0.05, an enrichment factor greater than 3.0, and a minimum count of 3.

Subsequently, we conducted protein–protein interaction (PPI) enrichment analysis along with the application of the Molecular Complex Detection (MCODE) algorithm to discern densely interconnected network modules among the 63 proteins of interest. This was achieved using STRING (available at https://string-db.org/), a web-based tool that aggregates, scores, and integrates PPI data from public repositories and augments it with computationally-derived functional predictions^73^, to analyze the PPI network centered around KDELR1/2/3 proteins in the context of LUAD progression.

In order to mitigate the subjective limitations inherent in Gene Ontology (GO) and Kyoto Encyclopedia of Genes and Genomes (KEGG) analyses, we further performed grouped Gene Set Enrichment Analysis (GSEA) using the DESeq2 and enrichplot packages in the R programming language. This analysis drew upon gene sets from the Molecular Signatures Database v7.0, including Hallmark gene sets, C2(curated gene sets) and C6(oncogenic signatures gene sets), as well as whole transcriptome data from all LUAD samples within the TCGA database. The analysis was considered statistically significant when the results met |NES| > 1, NOM p-value < 0.05 and FDR q-value < 0.25.

### Immunocorrelation analysis

We evaluated the association between the expression levels of KDELRs and 22 kinds of infiltrating immune cells (including 3 subsets of B-cells, 7 types of T-cells, 2 kinds of NK cells, Monocytes, 3 classes of macrophages, 2 kinds of dendritic cells, 2 forms of Mast cells, Eosinophils, and neutrophils) together with tumor purity using the ggpubr and ggstatsplot packages for the R language.

Then we leveraged the TIMER database (https://cistrome.shinyapps.io/timer/) to establish associations between KDELRs and a series of immunological markers widely recognized as proxies for various immune cell types^74^. Additionally, we investigated the potential relationships between KDELRs and the immune checkpoint molecules programmed cell death protein 1 (PD1), programmed death-ligand 1 (PD-L1), and cytotoxic T-lymphocyte-associated protein 4 (CTLA-4), thereby contributing to the understanding of the immunological landscape and therapeutic implications in the context of KDELRs' expression in malignancies.

### Cell lines and clinical samples

The human LUAD A549 and the Bronchial Epithelium transformed with Ad12-SV40 2B (BEAS-2B) cells were obtained from the Institute of Translational Medicine, First Hospital of Jilin University. These cell lines were cultured in a dulbecco's modified eagle medium (DMEM) (Procell, Wuhan, China), enriched with 10% fetal bovine serum (Cellmax, Lanzhou, China) and 1% antibiotics (Penicillin/Streptomycin/Amphotericin B) at 37 °C in a cell culture incubator.

Furthermore, we collected paired cancer and adjacent non-cancerous tissues from 23 LUAD patients who underwent surgical resection at the First Hospital of Jilin University from June to October 2023. It is noteworthy that none of the patients had undergone any preoperative interventions such as radiation therapy, chemotherapy, or other systemic treatments prior to the surgery. Upon excision, all tissue samples were frozen with liquid nitrogen immediately, and then stored at – 80 °C.

### qRT-PCR

Quantitative reverse transcription polymerase chain reaction (qRT-PCR) was conducted to obtain mRNA expression data for KDELR1, KDELR2, and KDELR3, thereby constituting a validation dataset to substantiate our initial observations derived from the TCGA and GEO database analyses.

Total RNA was extracted from both tissue samples and cultured cells using TRIzol reagent (Takara, Shiga, Japan). Subsequently, RNA was reversely transcribed into complementary DNA (cDNA) employing the First Strand cDNA Synthesis Kit (yeasen, Shanghai, China). cDNA was amplified by qRT-PCR using the SYBR Green mix kit (GenStar, Beijing, China) on the ABI 7500 Real-Time PCR system, with the 2^−ΔΔCT^ method implemented for relative quantification. β-Actin was used as an internal reference for mRNA and lncRNA, while U6 was utilized as the internal control for miRNA. The primers sequences employed in these reactions are detailed in Supplementary Table S3.

### Statistical analysis

In processing the expression profile data downloaded from TCGA, the choice between the "Wilcoxon rank-sum test" or the "Welch's t-test" was made based on the characteristics of the data format to assess differences in gene expression. When comparing paired samples within TCGA, GEO databases and our institution, either the "Wilcoxon signed-rank test" or the "paired-sample t-test" was adopted depending on the nature of the grouped data formats. Other statistical computations were automatically derived from the online databases, where P-values less than 0.05 or log-rank p-values below 0.05 were considered statistically significant. Differences were considered to be significant if p < 0.05*; p < 0.01**; p < 0.001***.

### Ethics declarations

This study was performed in line with the principles of the Declaration of Helsinki. Approval was granted by the Ethics Committee of the First Hospital of Jilin University (2023-323). The informed consent was obtained from all participants and/or their legal guardians.

### Supplementary Information

Supplementary Figure S1.

Supplementary Figure S2.

Supplementary Figure S3.

Supplementary Figure S4.

Supplementary Table S1.

Supplementary Table S2.

Supplementary Table S3.

## Data Availability

The data that support the findings of this study are available from the corresponding author upon reasonable request.

## Abbreviations

AnimalTFDB: Animal transcription factor database
BEAS-2B: Bronchial epithelium transformed with Ad12-SV40 2B
CPTAC: Clinical proteomic tumor analysis consortium
ceRNA: Competing endogenous RNA
cDNA: Complementary DNA
CTLA-4: Cytotoxic T-lymphocyte-associated protein 4
DFS: Disease-free survival
DMEM: Dulbecco's modified eagle medium
ER: Endoplasmic reticulum
EGA: European genome-phenome archive
FP: First progression survival
GEO: Gene expression omnibus
GO: Gene ontology
GSEA: Gene set enrichment analysis
GTEx: Genotype-tissue expression
HPA: Human protein atlas
K-M: Kaplan Meier
KEGG: Kyoto encyclopedia of genes and genomes
LUAD: Lung adenocarcinoma
KDELR: Lys-Asp-Glu-Leu receptor
MCODE: Molecular complex detection
NSCLC: Non-small cell lung cancer
OS: Overall survival
PD1: Programmed cell death protein 1
PD-L1: Programmed death-ligand 1
PPI: Protein–protein interaction
qRT-PCR: Quantitative reverse transcription polymerase chain reaction
RFS: Relapse-free survival
TCGA: The cancer genome atlas
TFs: Transcription factors
TIME: Tumor immune microenvironment
TICs: Tumor-infiltrating immune cells
TME: Tumor microenvironment
