# Supplementary figures and images for: Exploring the potential biological significance of KDELR family genes in lung adenocarcinoma
Source: Sci Rep. 2024 Jun 27;14:14820. doi: 10.1038/s41598-024-65425-2 (PMC11211404; doi:10.1038/s41598-024-65425-2)

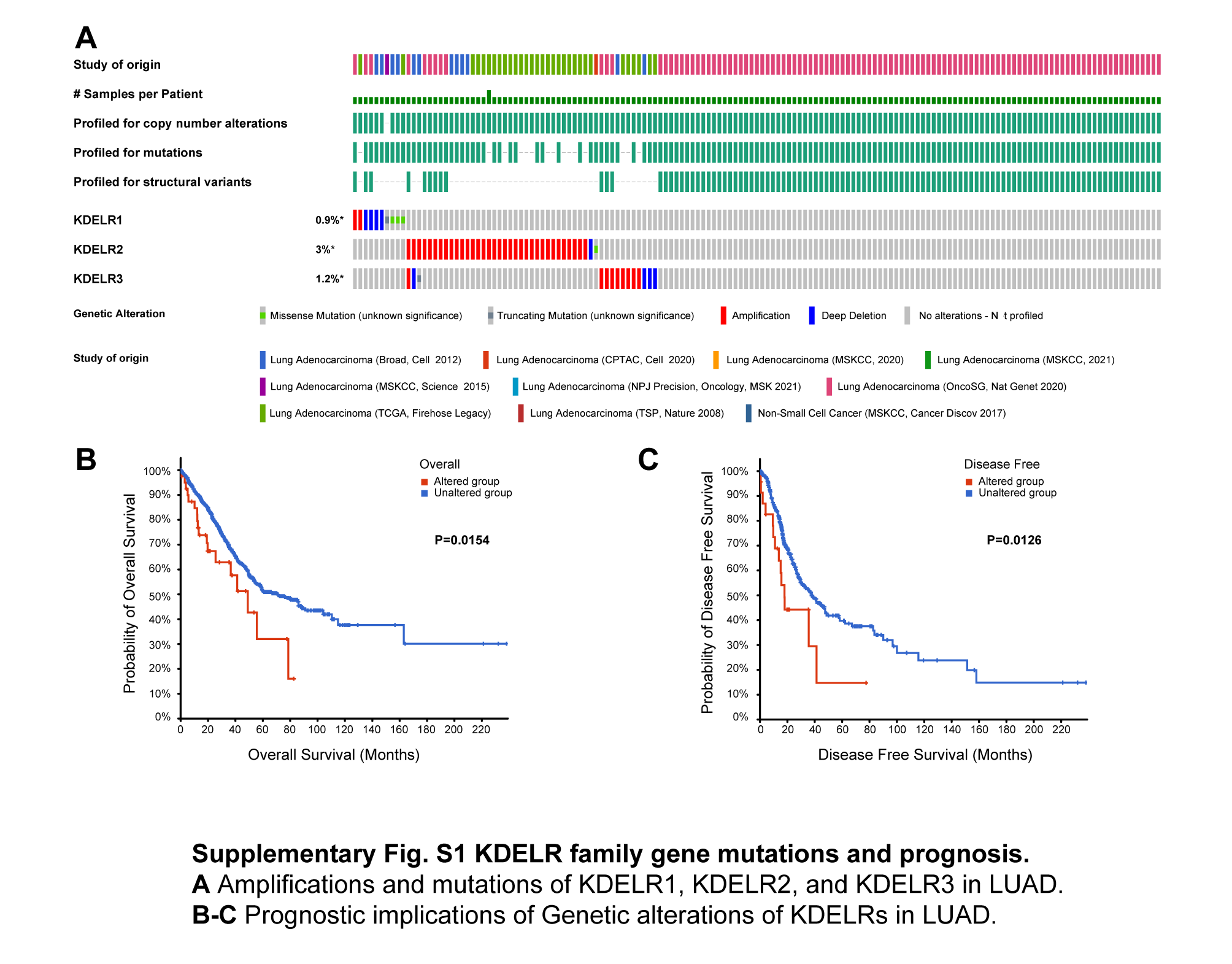

Supplement: Supplementary file 1 — Supplementary Figure S1. [file 41598_2024_65425_MOESM1_ESM.tif]

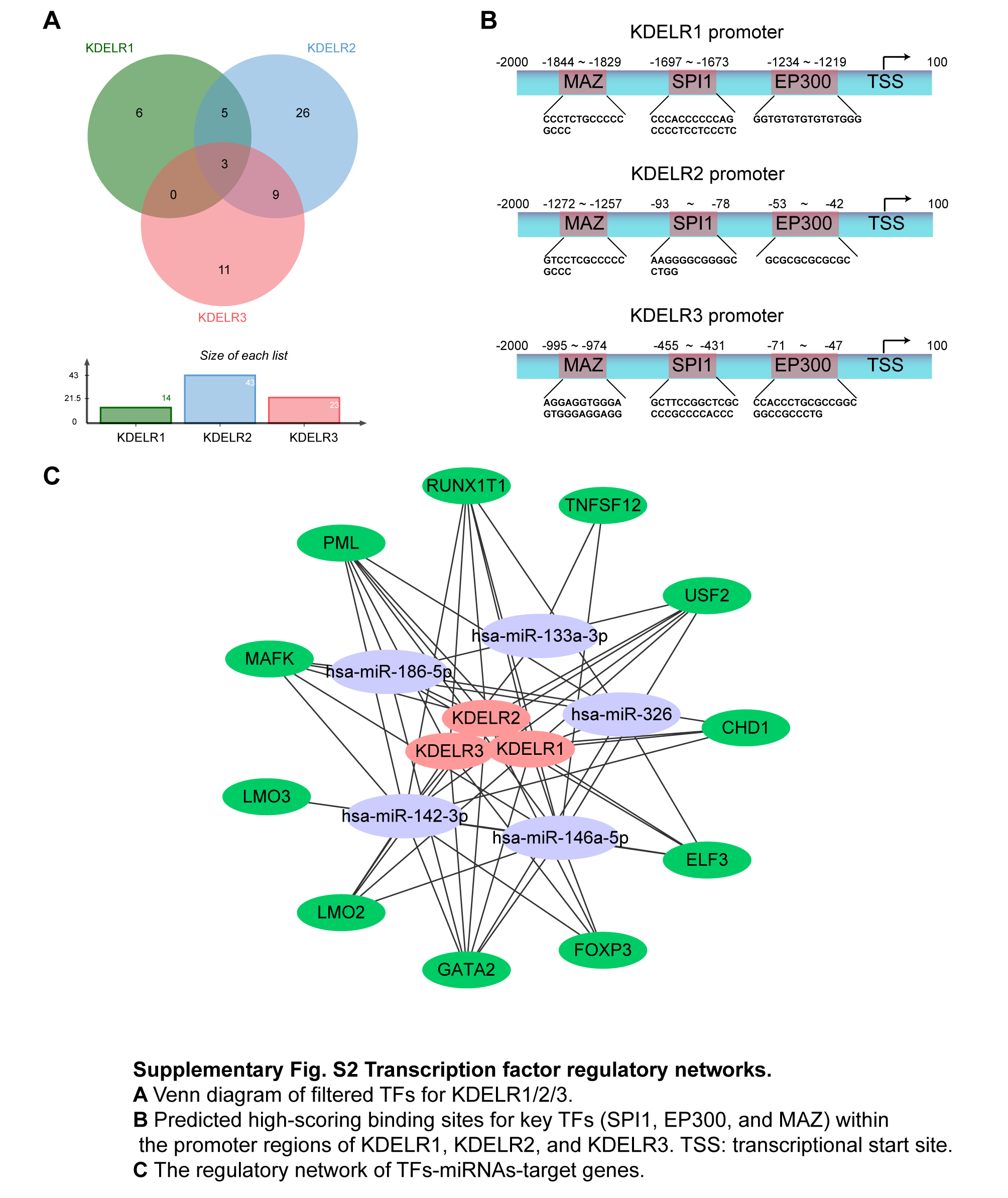

Supplement: Supplementary file 2 — Supplementary Figure S2. [file 41598_2024_65425_MOESM2_ESM.tif]

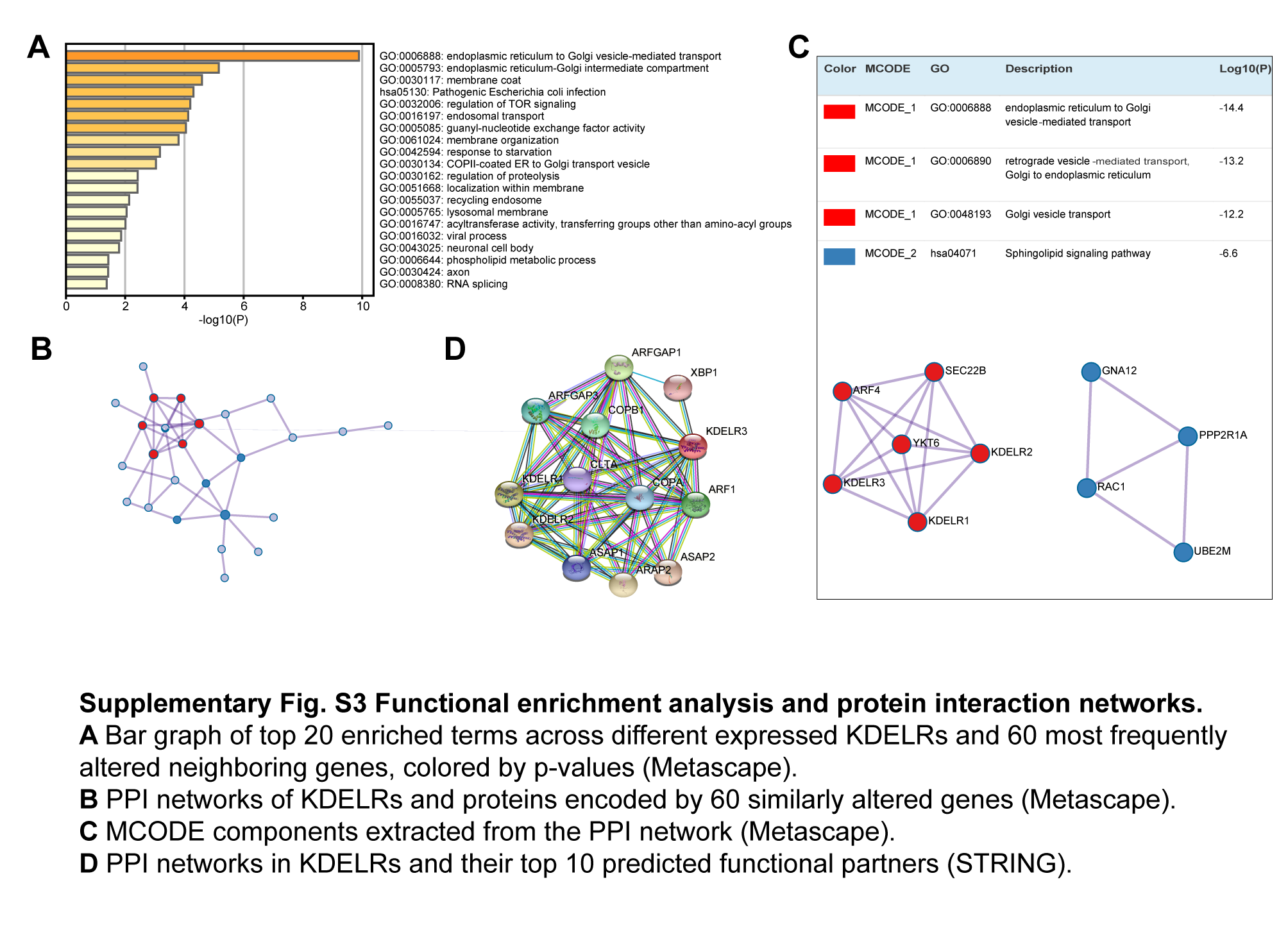

Supplement: Supplementary file 3 — Supplementary Figure S3. [file 41598_2024_65425_MOESM3_ESM.tif]

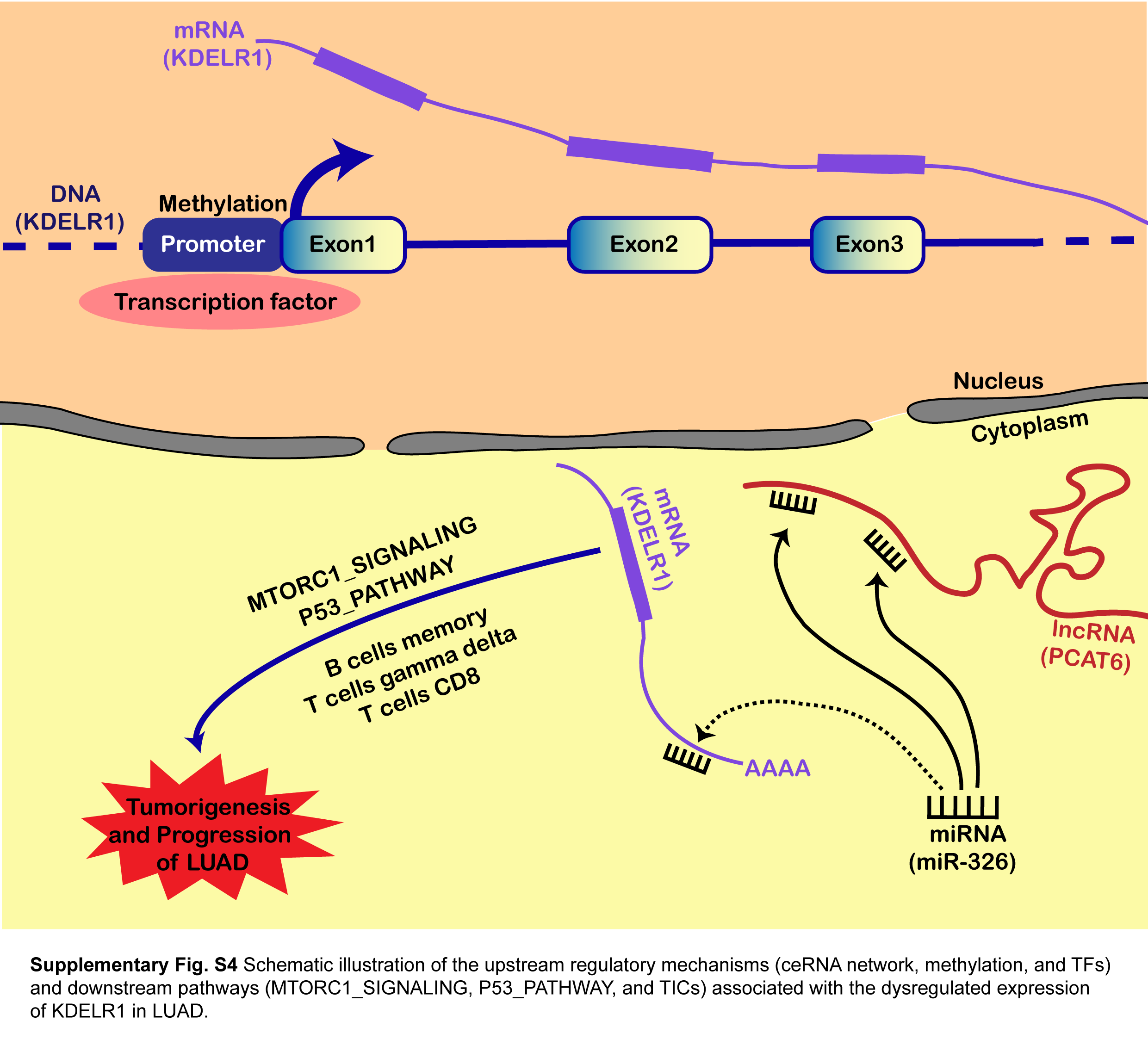

Supplement: Supplementary file 4 — Supplementary Figure S4. [file 41598_2024_65425_MOESM4_ESM.tif]
