# Supplementary material for: Exploring the potential biological significance of KDELR family genes in lung adenocarcinoma
Source: Sci Rep. 2024 Jun 27;14:14820. doi: 10.1038/s41598-024-65425-2 (PMC11211404; doi:10.1038/s41598-024-65425-2)
Supplement: Supplementary file 5 — Supplementary Table S1. [file 41598_2024_65425_MOESM5_ESM.docx]

**Supplementary Table S1.** **Prediction of miRNAs binding to KDELR1/2/3**

| **GeneName** | **miRNA name** | **Predicting program** | **Programs number** |
| --- | --- | --- | --- |
| KDELR1 | hsa-miR-326 | PITA,miRmap,miRanda,miRanda | 4 |
| KDELR1 | hsa-miR-146a-5p | PITA,miRanda,miRanda | 3 |
| KDELR1 | hsa-miR-331-3p | PITA,miRmap,TargetScan | 3 |
| KDELR1 | hsa-miR-146b-5p | PITA,miRanda | 2 |
| KDELR1 | hsa-miR-1271-5p | PITA,PicTar,TargetScan | 3 |
| KDELR1 | hsa-miR-96-5p | PITA,PicTar,TargetScan | 3 |
| KDELR1 | hsa-miR-224-5p | PITA,miRmap,miRanda,TargetScan | 4 |
| KDELR1 | hsa-miR-665 | PITA,miRmap,microT | 3 |
| KDELR1 | hsa-miR-182-5p | PITA,microT,miRanda,PicTar,TargetScan | 5 |
| KDELR1 | hsa-miR-185-5p | PITA,RNA22,,miRanda | 3 |
| KDELR1 | hsa-miR-330-5p | PITA,miRmap,miRanda | 3 |
| KDELR1 | hsa-miR-340-5p | PITA,microT,TargetScan | 3 |
| KDELR1 | hsa-miR-498 | PITA,microT | 2 |
| KDELR1 | hsa-miR-888-5p | PITA,microT | 2 |
| KDELR1 | hsa-miR-335-5p | PITA,miRanda,PicTar,TargetScan | 4 |
| KDELR2 | hsa-miR-142-3p | PITA,microT,miRanda,TargetScan | 4 |
| KDELR2 | hsa-miR-1-3p | PITA,miRmap,miRanda,TargetScan | 4 |
| KDELR2 | hsa-miR-186-5p | PITA,miRmap,microT,miRanda,TargetScan | 5 |
| KDELR2 | hsa-miR-149-5p | PITA,miRmap,microT,miRanda | 4 |
| KDELR2 | hsa-miR-410-3p | PITA,microT,miRanda | 3 |
| KDELR2 | hsa-miR-7-5p | PITA,miRmap,miRanda | 3 |
| KDELR2 | hsa-miR-329-3p | PITA,miRmap,miRanda | 3 |
| KDELR2 | hsa-miR-206 | PITA,miRmap,microT,miRanda,TargetScan | 5 |
| KDELR2 | hsa-miR-506-3p | PITA,microT,miRanda,TargetScan | 4 |
| KDELR2 | hsa-miR-655-3p | PITA,microT,TargetScan | 3 |
| KDELR2 | hsa-miR-520f-3p | PITA,miRmap,microT,TargetScan | 4 |
| KDELR2 | hsa-miR-548o-3p | PITA,miRmap,microT | 3 |
| KDELR2 | hsa-miR-495-3p | PITA,microT,miRanda | 3 |
| KDELR2 | hsa-miR-124-3p | PITA,microT,miRanda,TargetScan | 4 |
| KDELR2 | hsa-miR-31-5p | PITA,miRmap,microT,miRanda,TargetScan | 5 |
| KDELR3 | hsa-miR-133a-3p | PITA,microT | 2 |
| KDELR3 | hsa-miR-381-3p | PITA,miRanda | 2 |
| KDELR3 | hsa-miR-137 | PITA,microT,miRanda | 3 |
| KDELR3 | hsa-miR-490-3p | PITA,miRmap,miRanda | 3 |
| KDELR3 | hsa-miR-219a-2-3p | PITA,miRmap | 2 |
| KDELR3 | hsa-miR-449a | PITA,miRmap,miRanda | 3 |
| KDELR3 | hsa-miR-34c-5p | PITA,miRmap,miRanda | 3 |
| KDELR3 | hsa-miR-133b | PITA,microT | 2 |
| KDELR3 | hsa-miR-19a-3p | PITA,microT,miRanda | 3 |
| KDELR3 | hsa-miR-449b-5p | PITA,miRmap,miRanda | 3 |
| KDELR3 | hsa-miR-488-3p | PITA,miRmap,miRanda,TargetScan | 4 |
| KDELR3 | hsa-miR-513b-5p | PITA,miRmap | 2 |
| KDELR3 | hsa-miR-4525 | miRmap,microT | 2 |
| KDELR3 | hsa-miR-154-5p | PITA,microT,miRanda | 3 |
| KDELR3 | hsa-miR-19b-3p | PITA,microT,miRanda | 3 |
