# Supplementary material for: Exploring the potential biological significance of KDELR family genes in lung adenocarcinoma
Source: Sci Rep. 2024 Jun 27;14:14820. doi: 10.1038/s41598-024-65425-2 (PMC11211404; doi:10.1038/s41598-024-65425-2)
Supplement: Supplementary file 6 — Supplementary Table S2. [file 41598_2024_65425_MOESM6_ESM.docx]

**Supplementary Table S2. Correlation analysis between KDELR family members and markers of immune cells**

| **Cell type** | **Gene marker** | **KDELR1** | | | | **KDELR2** | | | | **KDELR3** | | | |
| --- | --- | --- | --- | --- | --- | --- | --- | --- | --- | --- | --- | --- | --- |
|  |  | **None** | | **Purity** | | **None** | | **Purity** | | **None** | | **Purity** | |
|  |  | **Cor** | **P** | **Cor** | **P** | **Cor** | **P** | **Cor** | **P** | **Cor** | **P** | **Cor** | **P** |
| CD8+ T Cell | CD8A | -0.13 | ** | -0.156 | *** | -0.116 | ** | -0.117 | ** | -0.103 | * | -0.17 | *** |
|  | CD8B | -0.12 | ** | -0.131 | ** | -0.123 | ** | -0.129 | ** | -0.128 | ** | -0.174 | *** |
| T cell  (general) | CD3D | -0.161 | *** | -0.199 | *** | -0.15 | *** | -0.161 | *** | -0.068 | 0.125 | -0.151 | *** |
|  | CD3E | -0.141 | ** | -0.182 | *** | -0.163 | *** | -0.176 | *** | -0.105 | * | -0.207 | *** |
|  | CD2 | -0.176 | *** | -0.214 | *** | -0.149 | *** | -0.155 | *** | -0.072 | 0.1 | -0.16 | *** |
| B cell | CD19 | -0.049 | 0.27 | -0.077 | 0.089 | -0.139 | ** | -0.153 | *** | -0.131 | ** | -0.223 | *** |
|  | CD20(KRT20) | -0.011 | 0.799 | -0.007 | 0.87 | 0.088 | * | 0.088 | 0.0505 | 0.168 | *** | 0.165 | *** |
|  | CD38 | -0.102 | * | -0.111 | * | -0.079 | 0.0746 | -0.073 | 0.105 | -0.123 | ** | -0.18 | *** |
|  | CD79A | 0.015 | 0.739 | -0.001 | 0.985 | -0.088 | * | -0.087 | 0.0522 | -0.09 | * | -0.165 | *** |
| M1 | COX2(PTGS2) | 0.246 | *** | 0.238 | *** | 0.274 | *** | 0.266 | *** | 0.196 | *** | 0.192 | *** |
|  | IRF5 | -0.036 | 0.421 | -0.041 | 0.366 | 0.098 | * | 0.118 | ** | 0.241 | *** | 0.214 | *** |
|  | NOS2 | 0.051 | 0.246 | 0.048 | 0.289 | -0.001 | 0.987 | 0.001 | 0.98 | 0.047 | 0.291 | 0.025 | 0.444 |
|  | ROS1 | -0.131 | ** | -0.137 | ** | -0.074 | 0.0937 | -0.071 | 0.116 | 0.139 | ** | 0.112 | * |
| M2 | MRC1 | -0.181 | *** | -0.18 | *** | -0.07 | 0.11 | -0.052 | 0.248 | 0.077 | 0.0824 | 0.052 | 0.251 |
|  | CD163 | -0.085 | 0.0535 | -0.089 | * | 0.033 | 0.449 | 0.056 | 0.212 | 0.078 | 0.0775 | 0.041 | 0.359 |
|  | VSIG4 | -0.171 | *** | -0.176 | *** | -0.055 | 0.215 | -0.03 | 0.51 | 0.062 | 0.157 | 0.038 | 0.395 |
|  | MS4A4A | -0.175 | *** | -0.183 | *** | -0.041 | 0.349 | -0.018 | 0.695 | 0.098 | * | 0.069 | 0.127 |
| TAM | CD80 | -0.165 | *** | -0.189 | *** | -0.022 | 0.612 | -0.016 | 0.726 | 0.062 | 0.163 | 0.014 | 0.76 |
|  | CCL2 | -0.043 | 0.327 | -0.057 | 0.207 | 0.156 | *** | 0.186 | *** | 0.138 | ** | 0.106 | * |
|  | CD68 | -0.098 | * | -0.109 | * | -0.035 | 0.425 | -0.016 | 0.718 | 0.097 | * | 0.061 | 0.175 |
|  | IL10 | -0.164 | *** | -0.187 | *** | -0.022 | 0.616 | -0.001 | 0.984 | 0.087 | * | 0.054 | 0.235 |

**Supplementary Table S2 (continue)**

|  |  | KDELR1 | | | | KDELR2 | | | | KDELR3 | | | |
| --- | --- | --- | --- | --- | --- | --- | --- | --- | --- | --- | --- | --- | --- |
|  |  | None | | Purity | | None | | Purity | | None | | Purity | |
| Cell type | Gene marker | Cor | P | Cor | P | Cor | P | Cor | P | Cor | P | Cor | P |
| Neutrophl | CD15(FUT4) | 0.125 | ** | 0.118 | ** | 0.233 | *** | 0.224 | *** | 0.187 | *** | 0.17 | *** |
|  | MPO | -0.086 | 0.0524 | -0.083 | 0.0664 | -0.032 | 0.469 | -0.018 | 0.696 | -0.011 | 0.809 | -0.033 | 4.62E-01 |
|  | CD66b(CEACAM8) | -0.09 | * | -0.082 | 0.0694 | -0.11 | * | -0.102 | * | 0.009 | 0.841 | 0.004 | 0.937 |
|  | CD11b(ITGAM) | -0.092 | * | -0.097 | * | 0.005 | 0.903 | 0.032 | 0.477 | 0.11 | * | 0.07 | 0.121 |
|  | CCR7 | -0.096 | * | -0.124 | ** | -0.134 | ** | -0.141 | ** | -0.065 | 0.14 | -0.153 | *** |
| Monocyte | CD14 | -0.107 | * | -0.118 | ** | 0.021 | 0.631 | 0.05 | 0.268 | 0.078 | 0.0775 | 0.041 | 0.365 |
|  | CD115(CSF1R) | -0.083 | 0.0597 | -0.093 | * | 0.015 | 0.73 | 0.036 | 0.425 | 0.106 | * | 0.066 | 0.144 |
| Nature killer | KIR2DL1 | -0.05 | 0.253 | -0.048 | 0.286 | -0.192 | *** | -0.197 | *** | -0.228 | *** | -0.255 | *** |
| cell | KIR2DL3 | -0.064 | 0.146 | -0.071 | 0.113 | -0.081 | 0.0655 | -0.072 | 0.112 | -0.159 | *** | -0.183 | *** |
|  | KIR2DL4 | -0.043 | 0.331 | -0.061 | 0.178 | 0.009 | 0.84 | 0.007 | 0.881 | -0.09 | * | -0.127 | ** |
|  | KIR3DL1 | -0.005 | 0.917 | -0.009 | 0.838 | -0.133 | ** | -0.13 | ** | -0.107 | * | -0.13 | ** |
|  | KIR3DL2 | -0.088 | * | -0.095 | * | -0.103 | * | -0.103 | * | -0.135 | ** | -0.175 | *** |
|  | KIR2DS4 | -0.055 | 0.213 | -0.055 | 0.22 | -0.076 | 0.0856 | -0.065 | 0.153 | -0.111 | * | -0.142 | ** |
| Dendritic cell | HLA-DPB1 | -0.227 | *** | -0.249 | *** | -0.138 | ** | -0.133 | ** | -0.002 | 0.961 | -0.054 | 0.228 |
|  | HLA-DQB1 | -0.119 | ** | -0.125 | ** | -0.051 | 0.248 | -0.038 | 0.396 | 0.061 | 0.165 | 0.023 | 0.607 |
|  | HLA-DRA | -0.264 | *** | -0.286 | *** | -0.066 | 0.136 | -0.051 | 0.255 | 0.048 | 0.297 | 0.004 | 0.922 |
|  | HLA-DPA1 | -0.239 | *** | -0.259 | *** | -0.073 | 0.1 | -0.055 | 0.221 | 0.037 | 0.404 | -0.004 | 0.938 |
|  | BDCA-1(CD1C) | -0.147 | *** | -0.153 | *** | -0.09 | * | -0.079 | 0.0786 | 0.114 | ** | 0.082 | 0.0687 |
|  | BDCA-4(NRP1) | 0.056 | 2.07E-01 | 0.052 | 2.52E-01 | 0.208 | *** | 0.221 | *** | 0.201 | *** | 0.193 | *** |
|  | CD11c(ITGAX) | -0.074 | 9.26E-02 | -0.095 | ** | -0.065 | 0.143 | -0.056 | 0.217 | 0.036 | 0.414 | -0.022 | 0.62 |

**Supplementary Table S2 (continue)**

|  |  | KDELR1 | | | | KDELR2 | | | | KDELR3 | | | |
| --- | --- | --- | --- | --- | --- | --- | --- | --- | --- | --- | --- | --- | --- |
|  |  | None | | Purity | | None | | Purity | | None | | Purity | |
| Cell type | Gene marker | Cor | P | Cor | P | Cor | P | Cor | P | Cor | P | Cor | P |
| Th1 | IFNG | -0.136 | ** | -0.147 | ** | -0.092 | * | -0.083 | 0.0666 | -0.116 | ** | -0.163 | *** |
|  | IL12RB2 | 0.018 | 0.685 | 0.01 | 0.82 | 0.119 | ** | 0.122 | ** | 0.012 | 0.792 | -0.012 | 0.791 |
|  | WSX1(IL27RA) | -0.024 | 0.584 | -0.032 | 0.477 | 0.102 | * | 0.126 | ** | 0.264 | *** | 0.249 | *** |
|  | STAT1 | -0.009 | 0.84 | -0.018 | 0.693 | 0.105 | * | 0.118 | ** | 0.046 | 0.301 | 0.009 | 0.843 |
|  | STAT4 | -0.139 | ** | -0.18 | *** | -0.006 | 0.897 | -0.005 | 0.914 | 0.109 | * | 0.047 | 0.301 |
|  | TNF | -0.068 | 0.123 | -0.07 | 0.121 | -0.014 | 0.759 | 0.014 | 0.762 | 0.049 | 0.268 | 0.007 | 0.885 |
|  | T-BET(TBX21) | -0.147 | *** | -0.183 | *** | -0.149 | *** | -0.154 | *** | -0.109 | * | -0.189 | *** |
| Th2 | STAT5A | -0.084 | 0.057 | -0.099 | * | -0.042 | 0.338 | -0.035 | 0.434 | 0.048 | 0.272 | -0.01 | 0.831 |
|  | STAT6 | -0.012 | 0.788 | -0.003 | 0.946 | -0.103 | * | -0.103 | * | -0.031 | 0.486 | -0.036 | 0.42 |
|  | IL13 | -0.072 | 0.102 | -0.064 | 0.158 | -0.109 | * | -0.101 | * | -0.041 | 0.353 | -0.064 | 0.159 |
| Th9 | IRF4 | -0.035 | 0.429 | -0.046 | 0.306 | -0.091 | * | -0.081 | 0.0724 | -0.098 | * | -0.173 | *** |
|  | PU.1(SPI1) | -0.114 | ** | -0.132 | ** | -0.097 | * | -0.086 | 0.0572 | 0.062 | 0.16 | 0.01 | 0.819 |
|  | TGFBR2 | -0.058 | 0.191 | -0.066 | 0.146 | 0.061 | 0.17 | 0.073 | 0.106 | 0.135 | ** | 0.107 | * |
| Th17 | IL17A | -0.075 | 0.0896 | -0.088 | * | -0.109 | * | -0.104 | * | -0.073 | 0.0968 | -0.098 | * |
|  | STAT3 | 0.152 | *** | 0.15 | *** | 0.158 | *** | 0.155 | *** | 0.055 | 0.213 | 0.048 | 0.283 |
| Th22 | AHR | 0.089 | * | 0.082 | 0.679 | 0.404 | *** | 0.414 | *** | 0.37 | *** | 0.356 | *** |
|  | CCR10 | 0.021 | 0.642 | 0.027 | 0.547 | 0.039 | 0.383 | 0.036 | 0.42 | -0.002 | 0.956 | 0.007 | 0.876 |
| Tfh | BCL6 | 0.081 | 0.0666 | 0.074 | 0.0992 | 0.041 | 0.357 | 0.035 | 0.441 | -0.029 | 0.505 | -0.056 | 0.213 |
|  | CXCR5 | -0.046 | 0.293 | -0.071 | 0.113 | -0.106 | * | -0.109 | * | -0.085 | 0.0529 | -0.172 | *** |
|  | ICOS | -0.157 | *** | -0.192 | *** | -0.079 | 0.0722 | -0.075 | 0.0941 | -0.009 | 0.838 | -0.083 | 0.0667 |
|  | IL21 | -0.103 | * | -0.096 | * | -0.058 | 0.189 | -0.038 | 0.405 | -0.099 | * | -0.131 | ** |

**Supplementary Table S2 (continue)**

|  |  | KDELR1 | | | | KDELR2 | | | | KDELR3 | | | |
| --- | --- | --- | --- | --- | --- | --- | --- | --- | --- | --- | --- | --- | --- |
|  |  | None | | Purity | | None | | Purity | | None | | Purity | |
| Cell type | Gene marker | Cor | P | Cor | P | Cor | P | Cor | P | Cor | P | Cor | P |
| Treg | CCR8 | -0.091 | * | -0.095 | * | 0.037 | 0.403 | 0.053 | 0.243 | 0.047 | 0.287 | 0.004 | 0.921 |
|  | FOXP3 | -0.028 | 0.533 | -0.037 | 0.418 | -0.013 | 0.763 | -0.002 | 0.969 | 0.028 | 0.521 | -0.029 | 0.521 |
|  | STAT5B | -0.015 | 0.737 | -0.021 | 0.641 | -0.066 | 0.133 | -0.077 | 0.0892 | -0.122 | ** | -0.145 | ** |
|  | TGFB1 | 0.144 | ** | 0.133 | ** | 0.07 | 0.113 | 0.076 | 0.092 | 0.238 | *** | 0.198 | *** |

TAM Tumor-associated macrophage. Th T helper cell, Tfh Follicular helper T cell, Treg Regulatory T cell. None, Correlation without adjustment. Purity, Correlation adjusted by purity. Cor, R value of Spearman’s correlation. *P < 0.05; **P < 0.01; ***P < 0.001.
