# Supplementary material for: Exploring the potential biological significance of KDELR family genes in lung adenocarcinoma
Source: Sci Rep. 2024 Jun 27;14:14820. doi: 10.1038/s41598-024-65425-2 (PMC11211404; doi:10.1038/s41598-024-65425-2)
Supplement: Supplementary file 7 — Supplementary Table S3. [file 41598_2024_65425_MOESM7_ESM.docx]

**Supplementary Table S3. Primer sequences of qRT-PCR**

| **Primer** | **Forward primer (5’-3’)** | **Reverse primer (5’-3’)** |
| --- | --- | --- |
| KDELR1  KDELR2  KDELR3  PCAT6  β-Actin  Hsa-miR-326  Hsa-U6 | CACAGCCATTCTGGCGTTCCTG  CTCCTATGCCACAGTGTACC  GAGGCTGAGACCATAACTACTCAC  CCCCTCCTTACTCTTGGACAACACT  CAGGTCATCACCATTGGCAATGAG  ATCTGTCTGTTGGGCTGGAGGC  GTGCTCGCTTCGGCAGCACATATA | CCATGAACAGCTGCGGCAAGAT  ACCAGAAACTCCACTCGGAAG  TTTGTACTACTCCAGACACGACT  ACCGAATGAGGATGGAGACACCTA  CGGATGTCCACGTCACACTTCATGA  GAAGGGCCCAGAGGCGATCTGA  ATATGGAACGCTTCACGAATTTGCG |
